# Diversity in the Major Polysaccharide Antigen of *Acinetobacter Baumannii* Assessed by DNA Sequencing, and Development of a Molecular Serotyping Scheme

**DOI:** 10.1371/journal.pone.0070329

**Published:** 2013-07-29

**Authors:** Dalong Hu, Bin Liu, Lenie Dijkshoorn, Lei Wang, Peter R. Reeves

**Affiliations:** 1 TEDA School of Biological Sciences and Biotechnology, Nankai University, Tianjin, China; 2 The Key Laboratory of Molecular Microbiology and Technology, Ministry of Education, Tianjin, China; 3 Department of Infectious Diseases, Leiden University Medical Center, Leiden, The Netherlands; 4 Tianjin Research Center for Functional Genomics and Biochip, Tianjin, China; 5 School of Molecular Bioscience, University of Sydney, Sydney, Australia; Robert Koch Institut, Germany

## Abstract

We have sequenced the gene clusters for type strains of the *Acinetobacter baumannii* serotyping scheme developed in the 1990s, and used the sequences to better understand diversity in surface polysaccharides of the genus. We obtained genome sequences for 27 available serovar type strains, and identified 25 polysaccharide gene cluster sequences. There are structures for 12 of these polysaccharides, and in general the genes present are appropriate to the structure where known. This greatly facilitates interpretation. We also find 53 different glycosyltransferase genes, and for 7 strains can provisionally allocate specific genes to all linkages. We identified primers that will distinguish the 25 sequence forms by PCR or microarray, or alternatively the genes can be used to determine serotype by “molecular serology”. We applied the latter to 190 *Acinetobacter* genome-derived gene-clusters, and found 76 that have one of the 25 gene-cluster forms. We also found novel gene clusters and added 52 new gene-cluster sequence forms with different *wzy* genes and different gene contents. Altogether, the strains that have one of the original 25 sequence forms include 98 *A. baumannii* (24 from our strains) and 5 *A. nosocomialis* (3 from our strains), whereas 32 genomes from 12 species other than *A. baumannii* or *A. nosocomialis*, all have new sequence forms. One of the 25 serovar type sequences is found to be in European clone I (EC I), 2 are in EC II but none in EC III. The public genome strains add an additional 52 new sequence forms, and also bring the number found in EC I to 5, in EC II to 9 and in EC III to 2.

## Introduction

The genus *Acinetobacter* belongs to the Family *Moraxellaceae* in the Order *Pseudomonadales* of the *Gammaproteobacteria*, and comprises 30 named species [1] (http://www.bacterio.cict.fr/). Several presumptive additional species have also been described on the basis of DNA-DNA hybridization [2]. Most *Acinetobacter* species are environmental, but *Acinetobacter baumannii* and, to some extent, the closely related species *A. pittii* (genomic species 3) and *A. nosocomialis* (genomic species 13TU) have emerged as important nosocomial pathogens being notorious for their multidrug resistance and epidemic potential [2] [3], and are referred to as the *A. baumannii* complex. Many outbreaks of *Acinetobacter* are associated with three major clones, the European clones I–III [4] [5], which are now known to occur worldwide.

The first reports of surface polysaccharides for *Acinetobacte*r were for *A. venetianus* strain RAG-1, which has been studied in detail. RAG-1 was reported in 1972 [6] to produce an emulsifying agent, that is now known as emulsan, and the polysaccharide component was reported to be composed of two major sugars (D-galactosamine and an unidentified amino uronic acid), one minor sugar (D-glucose), and an unidentified fatty acid ester [7]. The gene cluster for synthesis of emulsan was identified [8] and has *wza*, *wzb* and *wzc* genes as a group at one end of the gene cluster and also *wzx* and *wzy* genes within the gene cluster, where they are intermingled with genes for synthesis of repeat unit intermediates and glycosyltranferase (GT) genes that were not further characterised [8].

**Figure 1:**
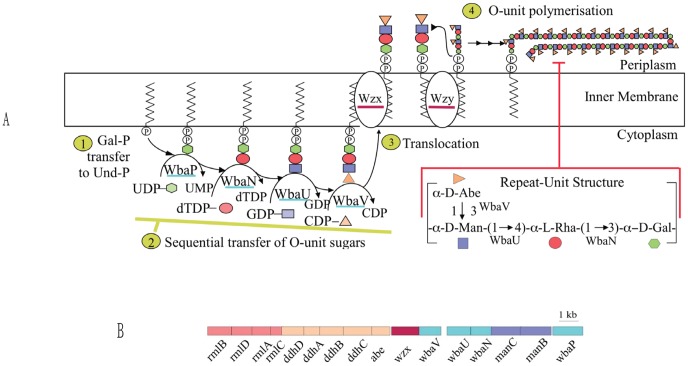
Biosynthesis of the O antigen of *S. enterica* group B. A. **Step 1**. Transfer of Gal-P to Und-P to give UndPPGal by Initial Transferase WbaP. **Step 2**. Sequential transfer of Rha, Man and Abe to complete the repeat unit. **Step 3**. Translocation of the UndPP associated repeat unit across the membrane. **Step 4**. Polymerisation of the repeat unit by Wzy. Note that the most recently added repeat unit is attached to the lipid UndPP component. Not shown is ligation to lipid-A/core and export to the outer membrane of the completed LPS molecules. **B.** Genetic map showing the genes for these steps [10]. Pathway genes and products are colour coded for each nucleotide sugar precursor.

**Figure 2:**
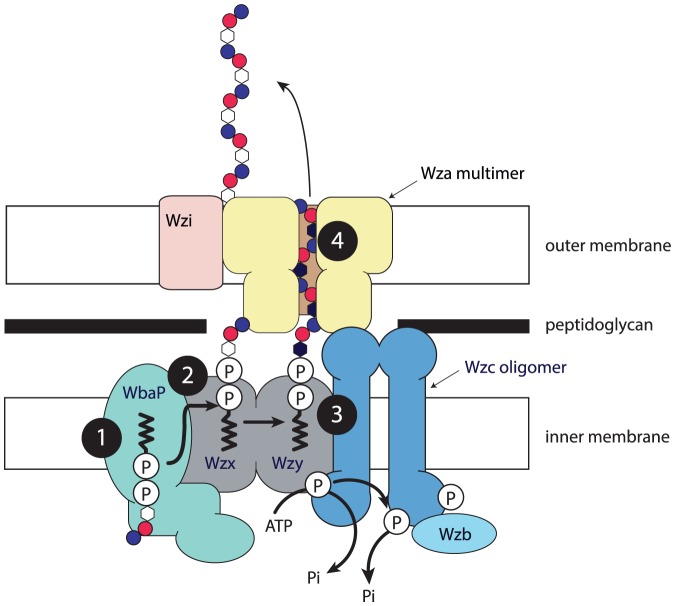
A model for the envelope-spanning enzyme complex involved in *E. coli* group 1 capsule assembly. Steps 1-3 overlap those in Figure 1 and cover repeat-unit assembly, translocation and polymerisation. Note that there is currently no experimental evidence for association of the IT, GTs, Wzx or Wzy with the well-documented Wza, Wzb, Wzc complex (Chris Whitfield, pers. comm.), which carries out Step 4, being export of the polymer to the cell surface. However the juxtaposition shows very well the flow of the monomer across the membrane to polymerisation and export. (Figure kindly provided by Chris Whitfield) [11] [12].

The presence of *wzx* and *wzy* genes tells us that emulsan is made by the Wzx/Wzy pathway [9] (Fig. 1). Before discussing the *Acinetobacter* gene clusters and polysaccharide structures, we look at synthesis of the *Salmonella enterica* group B O-antigen as a model for the Wzx/Wzy pathway [10] (Fig. 1), and export of the *Escherichia coli* K30 capsule (Fig. 2) as a model for the *wza*, *wzb*, *wzc* export pathway [11] [12].

The *S. enterica* group B repeat unit is synthesised in 4 steps starting with transfer of galactose phosphate (Gal-P) to a lipid carrier, undecaprenol phosphate (UndP), on the inner face of the cell membrane by initial transferase (IT) WbaP (Fig. 1. Step 1). This is followed by addition of other sugars by glycosyltransferases (GTs) (Step 2), and the complete RU is then translocated across the membrane to its periplasmic face by the Wzx translocase (Step 3). Each sugar residue is represented by a different symbol and colour, also applied to genes and proteins related to those sugars. The repeat units are then polymerised by Wzy (Step 4) to give the complete O antigen. The group B gene cluster is shown in Fig. 1B. It includes the *rml* and *man* sets of genes for synthesis of dTDP-rhamnose and GDP-mannose, and also the *ddh* set of genes that, together with the *abe* gene, code for the synthesis of CDP-abequose. The IT gene *wbaP* and the 3 genes for the GTs shown in Fig. 1A are also present, as are the *wzx* and *wzy* genes. In *S. enterica* the complete O antigen is ligated to lipidA/core by the WaaL ligase (not shown), encoded by the *waaL* gene in LPS core the gene cluster. The complete LPS is exported to the outer membrane by the Lpt complex [13] (not shown).

The model system for study of *wza*, *wzb* and *wzc* genes is the *E. coli* K30 capsule [11], which like *A. venetianus* RAG-1 also has *wzx* and *wzy* genes in the gene cluster. This indicates that polymer synthesis involves the Wzx/Wzy pathway [9] as discussed above. The Wza, Wzb and Wzc proteins form a complex (Fig. 2), with Wza embedded in the outer membrane, Wzc embedded in the inner membrane and Wzb associated with Wzc. The complex accepts the polymer made by Wzy and transports it to the cell surface, where it is thought to be attached to the surface, although details are not yet known [11]. The *E. coli* K30 repeat polymer can also be added to lipopolysaccharide (LPS) lipid A/core, when it becomes an O antigen, and this form of LPS is known as K_LPS_ [11]. Formation of K_LPS_ does not require Wza, Wzb or Wzc, but attachment to lipid A/core requires the action of WaaL ligase, encoded by the *waaL* gene in the LPS core gene cluster.

Coming back to *Acinetobacter*, Nakar et al [8] were able to attribute function to many of the *A. venetianus* genes. However the picture is complicated by a later report [14] that the exopolysaccharide (EPS) component of emulsan contains only galactosamine.

Further experiments on the *A. venetianus wee* gene cluster were carried out later involving mutations in *wzc*, deletion of *wzc* or *wzy*, replacement of the *wza*, *wzb*, *wzc* genes with the corresponding *E. coli* capsule K30 genes, or deletion of the whole gene cluster [15], [16], [17], [18]. Each of these either affected the pattern of chain length distribution or, in the case of the gene-cluster deletion, blocks emulsan synthesis completely, confirming that the *wee* gene cluster is responsible for synthesis of emulsan exopolysaccharide.

More recently the gene cluster for a capsule named K1 of strain AB307-0294 was shown to map to the same locus, as mutations in either *wza* or *wzc* blocked expression [19], and in 2011 the locus was recognised as one of several loci in *Acinetobacter* genomes that varied between genomes, in this case seen as coding for O-antigen biosynthesis [20].

There is also information on surface polysaccharides of *Acinetobacter lwoffii* strain F78, which has rough LPS (no O antigen) [21] and also a capsule [22]. The capsule has a repeat unit of 3 sugars, being L-FucNAc, D-QuiNR^1^4NR^2^ and L-GlcNR^3^3NR^4^A, where the 2 N-linked substituents on D-QuiN4N (R^1^ and R^3^) are one each of 3-HBA and alanine, and those on LGlcN3N (R^2^ and R^4^) are one each of 3-HBA or acetyl moieties.

A serotyping scheme for *A. baumannii* was developed by Traub from the late 1980s onward with 38 serovars in the last publication on the scheme in 2000 [23]. It was based on a polysaccharide that was generally referred to as O antigen, although Traub had noted [24] that “it is currently unknown whether the partially heat-resistant antigens involved represent lipopolysaccharide or microcapsular moieties”.

There have been structures published for 12 of the Traub serovar strains [25], but no genetic or biochemical studies, and there have been conflicting reports on other strains regarding the status of *A. baumannii* polysaccharides. In most cases the name capsule or O-antigen was applied based on method of extraction, and not a clear demonstration that the polysaccharide was indeed capsule or O antigen. Fregolino et al. [26] give a brief summary of these studies and refer to two cases of rough LPS (no O antigen) [27], [28] and one of smooth LPS (with O antigen) [29] in *A. baumannii*, and also two clearly defined capsules in *Acinetobacter* [30] [22], neither from *A. baumanni*. They themselves determined the structure of two *A. baumannii* capsules. The serovar-specific polysaccharides produced by the different serovars were originally referred by to Traub as O antigens (O1, O10 etc), and the structures were published as O-antigen structures [25]. However given the current uncertainty on the status of the polysaccharide, and the possibility that some or all may be expressed as either capsule or O antigen, we will use the term serovar (Sv) when discussing serology, and will refer to the gene clusters as respective polysaccharide gene clusters (PSgc) using corresponding numbering, e.g. Sv1 and PSgc1, etc. This is not only because of the uncertainty regarding the location of the polysaccharide, but also because for new gene clusters identified by sequence, we will have only sequence data, and no structural or serological data.

Serotyping using the Traub system and a system based on monoclonal antibodies against certain O antigens developed in the 1990s [31] were not widely used because, since the 1990s, a variety of genotyping methods have become available for epidemiologic typing of *Acinetobacter* strains. However, antigenic variation is an important factor in pathogenicity and adaptation of clones, and has been the basis for definition of clones within a number of species. Perhaps the best-known example is the O157:H7 clone of *E. coli* [32]. Despite the development of the serotyping scheme in the 1990s by Traub and the work of Pantophlet et al. [31], which were major contributions to knowledge of antigenic variation of *Acinetobacter*, there is currently no accepted typing system for *A. baumannii* to compare with the Kaufman-White scheme for *Salmonella* and similar schemes for *E. coli* [33], *Streptococcus pneumoniae* [34] and other major pathogens.

In this paper we report the sequences of the gene clusters for the Traub scheme Sv1 to Sv27 type strains [35] that are still available, and propose a sequence-based molecular typing scheme based on the variation in these polysaccharide gene clusters.

## Materials and Methods

### Strains

The 27 Sv type strains were originally from the WH Traub collection at the Institut fur Medizinische Mikrobiologie und Hygiene, Universitat des Saarlandes and sent to SG Wilkinson (School of Chemistry, University of Hull), who sent them to L. Dijkshoorn. All were identified originally as *A. baumannii* by phenotypic methods, which are not sufficient in the light of the current taxonomy, and we find some to be *A. nosocomialis* (see below).

### Sequencing

Whole genome sequencing of 27 isolates was performed with Solexa pair-end sequencing technology [36]. The Solexa Genome Analyzer IIx (Illumina, Little Chesterford, Essex) was used to sequence each isolate to a depth of between 90 and 100-fold coverage. The Illumina data were *de novo* assembled using VelvetOptimiser v2.2 (http://bioinformatics.net.au/software.velvetoptimiser.shtml). Gaps within the gene clusters for the major polysaccharide antigen were closed by directed PCR and the products sequenced with BigDye terminator chemistry on ABI 3730 capillary sequencers. Accession numbers for the whole genomes and gene clusters are given in Table S1.

Genes were first identified by BLAST searches and then subjected to further analysis for confirmation or clarification. For sugar pathway genes a BLAST search against the UniProt/SwissProt database was used to confirm allocation of the genes by pathway (Table S2). All of the *wzx* genes coded for proteins with 12 transmembrane segments as expected [9] and *wzy* genes coded for proteins with 11 transmembrane segments and the expected periplasmic loop [9]. Most GT genes belonged to pfam families Gly_transf_sug(PF04488), Glyco_transf_52(PF07922), Glycos_transf_1(PF00534) or Glycos_transf_2(PF0053). Four of them (*wafW*, *wagB*, *wagT* and *wagV*) had no pfam family but had good BLAST hits to other GTs [37].

### Classfication of homology groups

The *wzx*, *wzy* and GT genes were separately allocated to homology groups (HGs) using the program OrthoMCL v2.0 (http://orthomcl.org/common/downloads/software/v2.0/), and a 50% amino-acid identity level as the cut-off. With few exceptions, where an HG had more than one member, the sequences share high level identity, and the HGs corresponded to genes. In the case of GTs they were given gene names, but for *wzx* and *wzy* were given number subscripts as names (*wzx*_1) etc (Table S3).

### Extraction of polysaccharide gene clusters from genome sequences in databases

190 *Acinetobacter* genome sequences were downloaded from databases. Many of the genomes had draft sequences and for 25 the gene cluster was either not found or was too fragmented for analysis, so excluded from further analysis. We used the *wzy* gene to provisionally assign the remaining 165 gene-cluster sequences to a sequence form. The other genes in those gene clusters with a given sequence form allocation were then checked. For the 147 complete gene-cluster sequences, those with the same *wzy* HG were found to have the same gene-cluster-specific set of genes in the same order. The other 18 gene clusters had gaps, but were fully consistent with complete gene clusters with the same *wzy* HG, and were treated as members of the relevant gene-cluster forms. Seventy-six new strains were allocated to one of the 25 pre-existing gene-cluster forms and 89 genomes to 52 new gene-cluster forms.

### Generating a tree for the *Acinetobacter* genome sequences and identifying the EC I, EC II or EC III clones

We generated a tree using 6 house-keeping genes used in previous phylogenetic studies, being *cpn60*, *fusA*, *pyrG*, *recA*, and *rplB* used by Diancourt *et al.* [38], and *ompA* used by Turton *et al.* [39]. We used Clustalw v2.0 (http://www.ebi.ac.uk/Tools/msa/clustalw2/) to align the sequences, and then phyML v3.0 (http://www.atgc-montpellier.fr/phyml/) to build a maximum likelihood tree for 217 genomes, using the JC69 module. The EC I, EC II and EC III clones were located on the tree using MLST data from Diancourt *et al* [39].

## Results and Discussion

**Figure 3:**
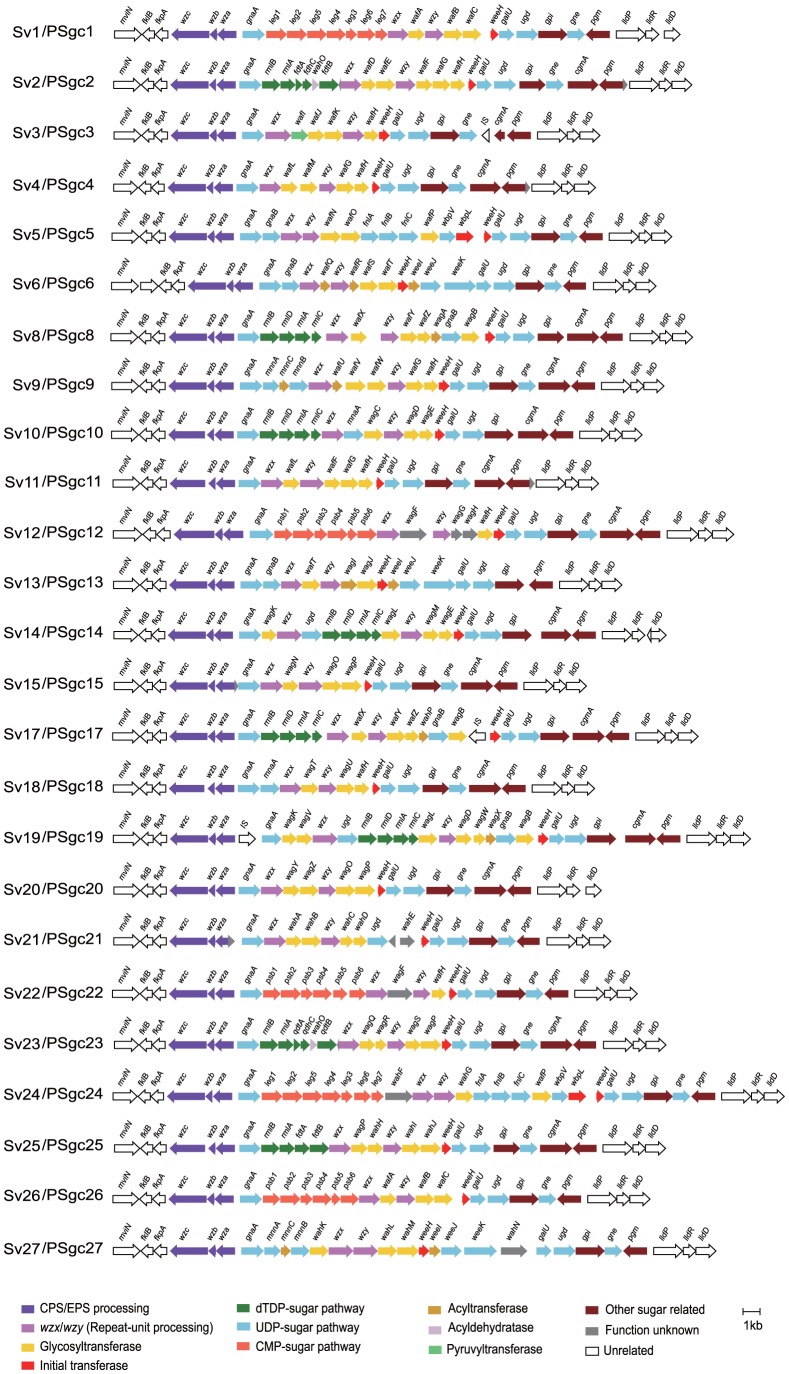
The polysaccharide gene clusters of the type strains of the *A. baumannii* complex serovars 1-27.

We obtained genome sequences of the type strains for serovars 1–27 of the Traub serotyping scheme being those that we found to be available (Table S1). Each genome has a gene cluster between the *fkpA* and *lldP* genes resembling that found in *A. venetianus* RAG-1 [8], with what appear to be two divergent operons, one on the left with *wza*, *wzb* and *wzc* genes transcribed in that order, and a much longer one on the right that includes genes for synthesis and processing of the repeat unit, plus a *pgm* gene transcribed separately (Fig. 3). There are also genes for synthesis of the lipid A/core component of LPS in all genomes, but no other gene cluster for surface polysaccharide synthesis in our genome sequences, and clearly this gene cluster is responsible for the major polysaccharide antigen, as was shown experimentally for emulsan of *A. venetianus* strain RAG-1.

Before discussing the details we need to clarify the situation regarding the species *A. baumannii*. The 27 strains [24] were received as *A. baumannii*, as originally classified by Traub by phenotypic methods. These do not allow for reliable identification of all *Acinetobacter* species, in particular the closely related, clinically important species *A. baumannii, A. pittii* (genomic species 3) and *A. nosocomialis* (genomic species 13TU) and the environmental species *A. calcoaceticus* sometimes referred to as the *A. calcoaceticus-A. baumannii* complex [3], [40] [41] [42]. We generated a tree based on the genome sequences of our strains and 190 publicly available genome sequences (Materials and Methods), and found that the Sv2, Sv4 and Sv11 strains are *A. nosocomialis* and the others are *A. baumannii* (Table S1).

There are two pairs of strains with the same genes and very little sequence difference, being Sv7/Sv9 and Sv16/Sv23. We therefore have only 25 gene cluster types for the 27 strains.

There are structures for both Sv16 and Sv23 [25], and the structure for Sv23 is fully compatible with the shared sequence, whereas that for Sv16 is much less compatible (see below). Strain LUH3714 is the representative strain for serovar 23 [25], and LUH3712 is the representative strain for serovar 16, so we have retained LUH3714 as the PSgc23 representative strain. It seems that the Sv16 structure was not done on the same strain that we have, and LUH3712 and LUH3714 are now treated as having the PSgc23 gene cluster.

Strain LUH5537 is the representative strain for serovar 7, and LUH5539 is the representative strain for serovar 9 [25]. The sequence shared by LUH5537 and LUH5539 is not consistent with the structure for Sv7 (see below), but there is no structure for Sv9. We opted to name the shared gene cluster PSgc9, with LUH5539 as the representative strain, and again it seems that the published Sv7 structure and sequence are not from the same strain.

We consider the 4 strains to be independent isolates as the LUH3712 and LUH3714 genome sequences differ at 48 sites, and the LUH5537 and LUH5539 genome sequences differ at 11. These sites are all in areas of good coverage and in our view the accumulation of these differences by mutation since isolation is most unlikely, and therefore it is not likely that the error involved one isolate getting 2 names, leading us to sequence the same strain twice. However there must have been strain mixups at some stage in the history of these strains. It is possible that the situation will be resolved, perhaps by finding more isolates for one or more of Sv7, Sv9, Sv16 and Sv23, but otherwise we propose that the designations PSgc7 and PSgc16 not be used for *Acinetobacter*.

Each of the gene clusters contains the *weeH* gene (Fig. 3, Table S2) that codes for an IT of the PHPT (**p**olyisoprenyl-phosphate **h**exose-1-**p**hosphate **t**ransferases) family [8], [43]. The best match among characterised PHPT genes is with *wbaP*, which was discussed above. The *weeH* gene is also present in all other *Acinetobacter* gene clusters that we know of and we will refer to them all as *wee* gene clusters. The PSgc5 and PSgc24 gene clusters had in addition a *wbpL* gene, which also codes for an IT but, as discussed below, we think that WbpL is not the IT for these structures. None of the gene clusters included a *waaL* gene for O-antigen ligation. A *waaL* homologue is present in *A. baylii* ADP1, but that was shown to be for protein glycosylation [44]. If any of the polysaccharides are present as an O-antigen there must be an alternative protein for the ligation reaction.

**Figure 4:**
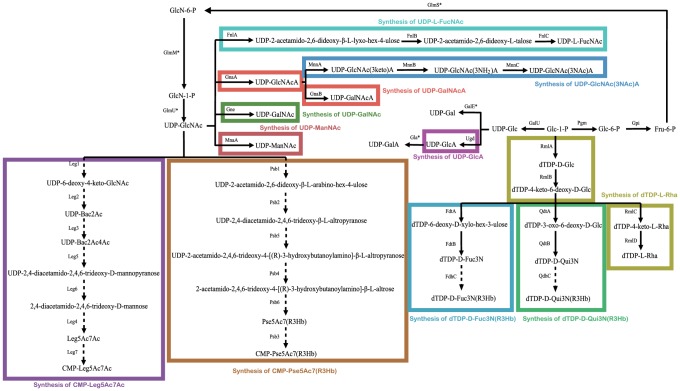
Biosynthesis pathways for sugars in *A. baumannii* major polysaccharides. Putative pathways are denoted by a broken line. Glc, D-glucose; GlcA, D-glucuronic acid; GlcN, 2-amino-2-deoxy-D-glucose; GlcNAc, 2-acetamido-2-deoxy-D-glucose; GlcNAcA, 2-acetamido-2-deoxy-D-glucuronic acid; Gal, D-galactose; GalA, D-galacturonic acid; GalNAc, 2-acetamido-2-deoxy-D-galactose; GalNAcA, 2-acetamido-2-deoxy-D-galacturonic acid; L-Rha, L-rhamnose(6-deoxy-L-mannose); ManNAc, 2-acetamido-2-deoxy-D-mannose; Fru, beta-D-fructose; L-FucNAc, 2-acetamido-2-deoxy-L-fucose; D-Fuc3N, 3-amino-3-deoxy-D-fucose; D-Fuc3N(R3Hb), 3-[(R)-3-hydroxybutanoylamino]-3-deoxy-D-fucose; D-Qui3N, 3-amino-3-deoxy-D-quinovose; D-Qui3N(R3Hb), 3-[(R)-3-hydroxybutanoylamino]-3-deoxy-D-quinovose; Pse5Ac7(R3Hb), 5-acetamido-3,5,7,9-tetradeoxy-7-[(*R*)-3-hydroxybutanoylamino]-L-*glycero-*L-*manno*-non-2-ulosonic acid (pseudaminic acid); Leg5Ac7Ac, 5,7-diacetamido-3,5,7,9-tetradeoxy-D-*glycero*-D-*galacto*-non-2-ulosonic acid (diacetyllegionaminic acid); Bac2Ac, 2-acetamido-4-amino-2,4,6-trideoxy-D-glucose (2-*N*-acetylbacillosamine); Bac2Ac4Ac, 2,4-diacetamido-2,4,6-trideoxy-D-glucose (*N,N′*-diacetylbacillosamine). *Genes found outside gene cluster.

There is a total of 11 sugars present in one or more of the 12 polysaccharide structures from *A. baumannii* strains of known serovar [25], being Gal, Glc, GlcNAc, GalNAc, GalNAcA, FucNAc, Fuc3N(*R*3Hb), Rha, ManNAc, Qui3N(*R*3Hb) and Leg5Ac7Ac. The first three are synthesised as UDP-linked sugars as part of the basic cell metabolism. The remaining eight have been identified in other gene clusters and require additional genes that are expected to be in the gene clusters. The genes for synthesis of GalNAc, GalNAcA, FucNAc, Rha, and ManNAc are well known and covered in a recent review [45]. There are recent descriptions of the CMP-Pse5Ac7(*R*3Hb) gene set in *Shigella boydii* O7 [45], the Leg5Ac7Ac gene set in *Legionella pneumophila* [46], and the Fuc3N(*R*3Hb) gene set in *E. coli* O103 [47]. The genes previously identified for these pathways are present in the appropriate gene clusters (Table S2, Text S1) and details of these pathways are shown in Fig. 4.

There is a set of genes in the PSgc12, PSgc22 and PSgc26 gene clusters that is very similar to the CMP-Pse5Ac7(*R*3Hb) gene set in *Shigella boydii* O7 [45]. There are structures for PSgc12 and PSgc22 but no sugar relating to these genes as discussed below. It appears that the PSgc12 and PSgc22 strains have the genetic capacity to make Pse5Ac7(*R*3Hb) but it is not expressed.

There is also a set of three genes in the PSgc7, PSgc9 and PSgc27 gene clusters that have 59%, 82% and 78%, amino acid identity to genes that code for synthesis of UDP-D-GlcNAc3NAc in *Pseudomonas aeruginosa* [48]. We have no structure for PSgc9 or PSgc27, and the reported Sv7 structure does not seem to relate to the PSgc7 sequence at all as discussed below. These genes are part of a five-gene pathway to D-Man(2NAc3NAm)A [49]. It appears that these serovars have the genetic capacity to make UDP- D-Glc(2NAc3NAc)A. The three genes have the species-related names *wbpBDE* in *P. aeruginosa* and *wlbBCD* in *Bordetella*. We propose that they be given the generic set of pathway-related names *mnnA*, *mnnB* and *mnnC* (**M**annose 2-**N**Ac 3**N**Ac), that can be applied to the genes in any species. The names *mnnD* and *mnnE* can be applied to genes for extension of the pathway to D-Man(2NAc3NAm)A, should they be found in other species.

There are also sugar pathway genes that are either always or generally present in the gene clusters. There is a *gnaA* gene after *wza* in all gene clusters. GnaA converts UDP-GlcNAc into UDP-GlcNAcA, not found in any of our structures. There is also a *gnaB* gene after the *gnaA* gene in three gene clusters, and at a different location in three others. GnaB converts GlcNAcA into GalNAcA, and both genes are present when GalNAcA is known to be present. The *galU*, *ugd*, *pgi*, *gne*, *cgmA* and *pgm* genes are present at the distal end of the gene cluster, always in the same order, and at the end a *pgm* gene in the opposite orientation. They were also found in the same arrangement in *A. venetianus* RAG-1, except for *cgmA*, which was also absent in 9 of our sequences. The *gne* gene is also missing in 6 of our sequences. Except for *gne*, these genes have functions in central metabolism and are not usually found in polysaccharide gene clusters. However *galU*, *ugd*, *pgi*, and *pgm* genes are required for components of all of the structures and it may be that with the lesser emphasis on sugar catabolism in *Acinetobacter*, their role in synthesis for the surface polysaccharide is relatively more significant and this may account for their location as part of the *wee* gene cluster. Further information on these genes is given in Text S2 [25] [50] [51] [52] [53].

The gene that we have called *gne* is usually annotated as *galE* in *Acinetobacter* genome sequences. GalE interconverts UDP-Gal and UDP-Glc, and the name Gne is used where the major or sole function is interconversion of UDP-GalNAc and UDP-GlcNAc. We have recently reviewed a range of *galE*-like 4-epimerase genes (Manuscript in revision with PLoS One) and find that although mostly annotated as *galE* based on BLAST searches, they fall into 4 clades, with *galE* and *gne* genes being in the same clade, and not readily distinguished on sequence alone. Most of our strains have two genes in the *galE/gne* clade, one of which is universally present outside of the gene cluster and associated with a *galM* gene. GalM catalyses the interconversion of α-D-galactose and β-D-galactose, and there is a *galM* gene in the *gal* operons of the *Enterobacteriaceae* [54]. The association with a *galM* gene supports the role of this gene as a UDP-Glc/UDP-Gal epimerase *galE* gene. The second *galE*-like gene is in the polysaccharide gene cluster, and we propose that it is the *gne* gene predicted to be present when the structure contains GalNAc, as do 9 of our 12 structures. It is common to find a *gne* gene in gene clusters for structures that include GalNAc, even in *Yersinia* species that have a *gal*-operon-encoded GalE epimerase that can use UDP-GlcNAc in addition to UDP-Glc as a substrate [55]. There are sometimes distinctive features for *gne* genes, but none that apply generally and it is not possible to predict from sequence the relative efficiencies for UDP-Glc and UDP-GlcNAc as substrates. Most of our structures include GalNAc, and the presence of a *galE* gene elsewhere makes *gne* a good prediction for this gene [56].

### Details of gene clusters for serovars with a reported polysaccharide structure

The PSgc-specific genes in each gene cluster, that are responsible for repeat-unit synthesis, lie between *wza* and *weeH*, both present in all of the gene clusters. *weeH* is followed by the *galU*, *ugd*, *pgi*, *gne*, *cgmA* and *pgm* genes that we discussed above.

**Figure 5:**
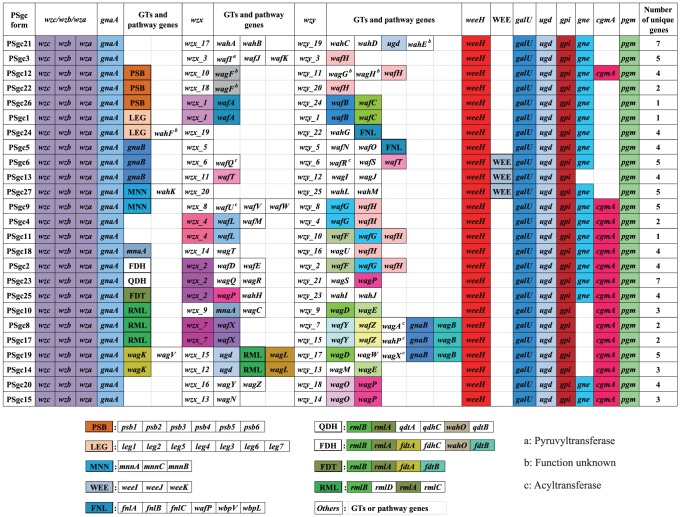
The pattern of gene variation in the 25 gene clusters. The genes for each cluster are presented in map order. Cells for genes present more than once are coloured. No colour means that the sequence is unique. Genes within a column with the same color have the same sequence. There are 5 genes (*gnaB*, *ugd*, *mnaA*, *wafT*, *wagP*) present at 2 loci within the gene clusters, so each in two columns and also given the same colour. Superscripts: .a: Pyruvyltransferase, b: Function unknown, c: Acyltransferase. Others are glycosyltransferases or pathway genes. The column “Number of unique genes” gives the number of GT and pathway genes plus *wzx* and *wzy* HGs that are unique to that PSgc.

**Figure 6:**
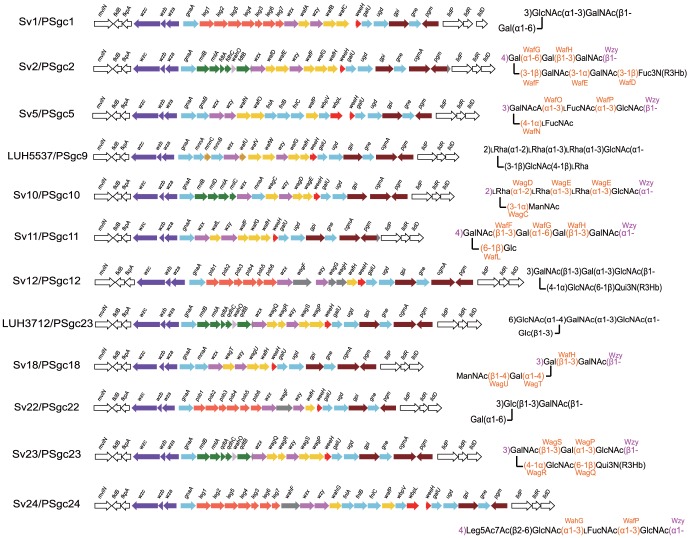
The gene clusters and structures for the serovars for which both are available. The gene names against some of the linkages are of GT genes proposed to be responsible for that linkage.

Each set of PSgc-specific genes includes a *wzx* and a *wzy* gene, but both are very variable in sequence (Fig. 5). Different Wzy polymerases make different polymerisation linkages as shown in Fig. 6 and are clearly functionally different. The *wzx* genes are almost as variable and, although this has only recently been recognised, we now believe that Wzx translocases have substantial specificity for the repeat unit that they translocate across the membrane [57]. We determined for both Wzy and Wzx the number of distinctive forms present using orthoMCL (see Materials and Methods) that identifies homology groups (HGs) with divergence levels that would commonly apply to different genes. Each of the 25 gene clusters had a unique Wzy HG and there were 20 Wzx HGs (see Table S3 and Fig. 5). Each of these central blocks also has several predicted GT genes, and in some cases nucleotide-sugar pathway genes for rare sugars. Each central block of genes constitutes a discrete serovar-specific set of genes as shown in Figs. 3, 5 and 6.

All of the structures include one or more of Gal, Glc and GlcNAc but as discussed above, the genes for synthesis of their precursors are present in all of the genomes so no further discussion is needed. Nine of the structures include GalNAc and there is a *gne* gene in each of the gene clusters, so again no further comment is needed. There are structures for 12 serovars [25] and the corresponding gene clusters are discussed below.

#### PSgc1

The structure has no rare sugars, but has a set of genes for synthesis of the CMP-linked precursor for Leg5Ac7Ac (Fig. 4, Text S2) that is not present in the structure. There are 3 sugars and so 2 GT linkages but 3 GT genes, which is one in excess of need. There are sufficient genes present for the structure, but it seems that there is a potential for a Leg5Ac7A side branch as the pathway genes are present and there is an extra GT gene. It remains for detailed analysis to allocate GT genes to the specific linkages and perhaps find a condition in which a side-branch Leg5Ac7A residue is added.

#### PSgc2

The PSgc2 structure includes a Fuc3N(*R*3Hb) structure and the required set of 5 genes (Fig. 4, Text S2) is also present. There are 5 GT linkages and 5 GT genes as expected so a perfect correlation between genes and structure.

#### PSgc5

The structure includes FucNAc and the 3 genes of the UDP-FucNAc pathway (Fig. 4, Text S2) are present. The structure also includes GalNAcA and the required *gnaB* gene (Fig. 4, Text S2) is present in the gene cluster. There are 3 linkages and 3 GT genes so again a perfect correlation.

Of particular interest is that the PSgc5 gene cluster not only includes the *fnlA,B,C* genes for synthesis of UDP- L-FucNAc but also *wbpV* and *wbpL*, both found in *P. aeruginosa* where *wbpL* codes for an IT for a range of O antigens, initiating repeat-unit synthesis with D-FucNAc or D-QuiNAc [58]. However this presence of a FucNAc residue and a putative FucNAc IT gene appears to be a coincidence. First it is it unlikely that an IT could have the capacity to add either the D or L- form of a sugar (by convention L-FucNAc is generally known as FucNAc). Secondly the data on the other GTs and the Wzy HGs (Tables S4 and S5) suggest that the initial sugar is in fact the GlcNAc residue. In particular the presence of a shared GT (WafP) suggests that it is responsible for the only shared linkage, the L-FucNAc (1-3) GlcNAc linkage that is the putative WbpL polymerisation linkage. Finally if L-FucNAc was the initial sugar, then the shared linkage would be the polymerisation linkage, but the *wzy* genes are in different HGs. In these circumstances it appears that the IT gene is *weeH* which is already implicated as a GlcNAc IT for PSgc10. The role of WbpV in *P. aeruginosa* is still not known [58], [59], and both *wbpL* and *wbpV* are without obvious function in PSgc5.

#### PSgc7

LUH5537, our serovar 7 representative, has a sequence that lacks the *rml* gene set whereas the structure includes Rha making the structure and gene cluster not consistent. The sequence also includes genes for D-GlcNAc3NAc, which is not present. As discussed above LUH5537 and LUH5539, the Sv9 representative, have the same sequence, and given the incompatibility of the PSgc7 structure and the LUH5537 sequence, we assume that the shared sequence codes for the PSgc9 structure, so that LUH5537 becomes a second PSgc9 sequence.

#### PSgc10

The structure includes Rha and ManNAc. The 4 genes of the dTDP-L-Rha gene cluster (Fig. 4, Text S2) are present as is the *mnaA* gene for UDP-ManNAc. There are four GT linkages, but only 3 GT genes. However there are 3 consecutive Rha residues and it is likely that one of the GTs add 2 Rha residues, as observed for consecutive Man residues in other repeat units [60]. Thus, there is a very good correlation between genes and structure.

#### PSgc11

The structure has no rare sugars and has 4 GT linkages and there are 4 GT genes, so there is a perfect correlation.

#### PSgc12

The PSgc12 structure has Qui3N(*R*3Hb) but the genes are not present, so the structure and gene cluster are not consistent. It does have the six genes for Pse5Ac7(R3Hb) (Fig. 4, Text S2), which is not in the structure.

#### PSgc16

LUH3712, our serovar 16 representative, has a gene cluster that is identical to that of LUH3714, the serovar 23 representative and, as discussed above, because the LUH3714 sequence is fully consistent with the structure, we opted to retain it as the serovar 23 representative strain. The Sv16 structure has no rare sugars and has 3 GT linkages and 4 GT genes so that would be consistent with the sequence, except that we know the linkages formed by the same set of genes for the Sv23 structure, and only the linkage proposed for WagR is also present in the Sv16 structure, so the fit is very poor. LUH3712 becomes a second PSgc23 strain.

#### PSgc18

The PSgc18 structure has ManNAc and the *mnaA* gene (Fig. 4, Text S2) is present. There are 4 linkages, and 4 GT genes are present, and therefore a perfect correlation.

#### PSgc22

The structure has no rare sugars and has 2 GT linkages. There is also a set of genes for CMP-Pse5Ac7(R3Hb) (Fig. 4, Text S2), as discussed above, that is not present in the published Sv22 structure. There is only 1 GT gene for two linkages, but there is a side-branch sugar and these are often added by transferases that map outside of the gene cluster, which may be the case here, so there is a reasonable correlation. An alternative possibility is that the missing GT gene is *wagF*, for which there is no predicted function and could represent a new GT family. The CMP-Pse5Ac7(R3Hb) set of genes is presumably not expressed as there is no Pse5Ac7(R3Hb) in the structure.

#### PSgc23

The structure includes Qui3N(*R*3Hb) and the gene set (Fig. 4, Text S2) is present. There are 4 linkages and 4 GT genes are present, so there is a perfect correlation between genes and structure.

#### PSgc24

The structure has both Leg5Ac7Ac and L-FucNAc and the gene sets for both (Fig. 4, Text S2) are present. There are 3 linkages but only 2 GT genes identified. There is an excellent correlation for sugar synthesis with 2 rare sugars present and the gene sets for both. The missing GT gene is probably *wahF*, for which there is no predicted function and, as for *wagF* in the PSgc22 gene cluster, could represent a new GT family.

As for PSgc5, the PSgc24 gene cluster not only includes the *fnlA,B,C* genes for synthesis of UDP-L-FucNAc (Fig. 4, Text S2) but also *wbpL* that codes for an IT that in *P. aeruginosa* initiates repeat-unit synthesis with D-FucNAc or D-QuiNAc. Also as for Sv5, the data on the other GTs and the Wzy HGs suggest that the initial sugar is in fact the GlcNAc residue. The same argument applies as for PSgc5, and *weeH* is the putative IT gene with GlcNAc as the first sugar. The PSgc5 and PSgc24 gene clusters have the same block of six genes, *fnlA*, *fnlB*, *fnlC*, *wafP*, *wbpV*, *wbpL*, that may have been acquired as a block. *wbpV* and *wbpL* occur in the same order in the *P. aeruginosa* O5 and O6 gene clusters. WbpV is proposed to be the 4-reductase involved in UDP-D-QuiNAc biosynthesis [49], and as for WbpL, there is no apparent role for WbpL in PSgc5 or PSgc24. The analysis of gene clusters in published genomes (see below) revealed a possible source for these genes. The six genes are part of a block of 8 genes (*GT2, fnlA, fnlB, fnlC, GT1, wbpV, wbpL, weeI*) also present in the new PSgc44 (see below). One can speculate that the six genes were acquired as a block from PSgc44, by recombination, but with only the *fnl* genes and *wafP* required for function. The original source was probably *Pseudomonas* as *wbpL* is the IT for all of the *P. aeruginosa* O antigens.

In summary the 12 polysaccharide gene clusters for which there is also a structure generally fit the reported structure very well. Seven of them (PSgc2, 5, 10, 11, 18, 23, 24) have a perfect or near perfect correlation between genes present and structure, and two (PSgc1 and PSgc22) have the expected genes but also have pathway genes for sugars that are not in the structure. Presumably there is a block somewhere in synthesis of the sugar, and it is not added. This is not unknown in other species and for example, the *S. enterica* group A O-antigen gene cluster has a 6-gene pathway for CDP-tyvlose synthesis but the final step requires the *tyv* gene, which is non-functional in group A. This means that it cannot synthesise tyvolose, which indeed is not in the structure, but as the defect is a frame-shift mutation in codon 4 of the gene, there is a long open reading frame and the failure is not apparent without experimental work [61]. Another example, for which we do not have the explanation, is the set of related gene clusters for *S. pneumoniae* capsule serogroups 15F, 15A, 15B and 15C. All four have *rmlB*, *rmlD*, and *glf* genes and a putative acetyl transferase gene that all appear to have no effect on the structure [62].

However three of the gene clusters are not consistent with the reported structures. The Sv7 and Sv16 strains have gene clusters that are identical to other gene clusters as discussed above, that better fit the structures, so the shared gene clusters have been named PSgc9 and PSgc23 respectively as discussed above. The PSgc12 gene cluster lacks genes for the Sv12 Qui3N(*R*3Hb) residue. In all three cases it appears that there have been errors in strain maintenance or transfer between labs, but we have used the name PSgc12 for our Sv12 strains, as we do not know if the sequence or structure is from the strain used for the serology.

### Allocation of GT genes to specific linkages

The putative GT genes from the 25 discrete sequences were allocated to homology groups (see Materials and Methods). This gave us 53 distinct GTs that were named as shown in Table S4A, Table S4B and Fig. 3. We also examined the variation in Wzy polymerases (Table S5), as a shared Wzy HG would indicate a shared or related polymerisation linkage, but there were no cases of shared Wzy HGs.

We were able to provisionally allocate 24 of the GT genes to specific functions based on homologies found in BLAST searches and presence of linkages shared by different polysaccharide structures, and also for seven of the Wzy HGs that were included in the analysis.

### Development of a molecular serotyping scheme

As expected each of the 25 gene clusters has a unique combination of genes, as they code for different structures. Each has a unique *wzy* gene, and 16 have a unique *wzx* gene (Table S3). Three *wzx* genes are found in 2 serovars, and another in 3 serovars. Eighteen of the serovars have from 1 to 4 unique GT genes, and there are 19 GTs that are present in from 2 to 7 serovars (Table S4C). These differences in the gene clusters are ideal for a diagnostic microarray, as where genes are shared there is usually very little variation. However a PCR-based scheme for identification could work well in the confines of a hospital if the major strains present had different polysaccharides. This is very likely outside of the major clones, and would provide a cheap and robust method for tracking strains, using perhaps the *wzy* gene and one PCR across a junction between 2 GT or pathway genes. We confirmed that PCR for *wzy* would work using the primers shown in Table S6. The primers were shown by BLAST searches to have no additional potential targets in any of the 27 strains, and worked well on the target strains. However the major clones may well not have enough serovar variation within a hospital to make molecular serotyping attractive unless there is a wide range of other strains present, but it would be appropriate if screening large numbers of strains that are not in the major clones. However it is now possible to extend the “serotyping” scheme using sequence data as shown below.

### Serotype determination of *Acinetobacter* strains based on genome sequences

We found 190 *Acinetobacter* genomes in public databases and found the polysaccharide gene cluster in 165 of them.

We found that the *wzy* gene-cluster sequences fell into 77 HGs, including the 25 already observed. We used the *wzy* HGs to provisionally assign each of the 165 gene clusters to one of 77 gene-cluster forms (**Materials and Methods**) and found that the gene clusters associated with each *wzy* HG also had a unique combination of genes, as expected if each is responsible for a different structure. We have given these gene clusters PSgc numbers only as we have not done any serology. However the strains are being distinguished by the gene clusters that generally determine serotypes, and there would probably be a very strong correlation. Seventy-six new strains were allocated to one of the 25 pre-existing named gene clusters. The 103 strains include 98 *A. baumannii* and 5 *A. nosocomialis* (3 from our own strains), with one not identified to species level. There are 51 new gene-cluster forms and these were named PSgc39–PSgc89 (Table S7). We did not use the numbers 28 to 38 as these properly belong to the serovars defined by Traub in 2000 [23], but for which we are not aware of any strains being available. However there remains the possibility that strains will be found. We added the *A. venetianus* RAG-1 gene cluster as PSgc90. There is now total of 77 PSgc identified for an *Acinetobacter* typing scheme.

In contrast to the *A. baumannii* complex genomes, there are 32 genome sequences from 12 species other than *A. baumannii* or *A. nosocomialis*, and none of them are in the originally defined 25 PSgc forms. The use of a sequence-based typing scheme enabled the addition of another 52 PSgc types. From a typing viewpoint sequence-based typing and serology are equally valid, but it remains to be seen how often they do not coincide.

### PSgc variation in the EC I, EC II and EC III clones

**Figure 7:**
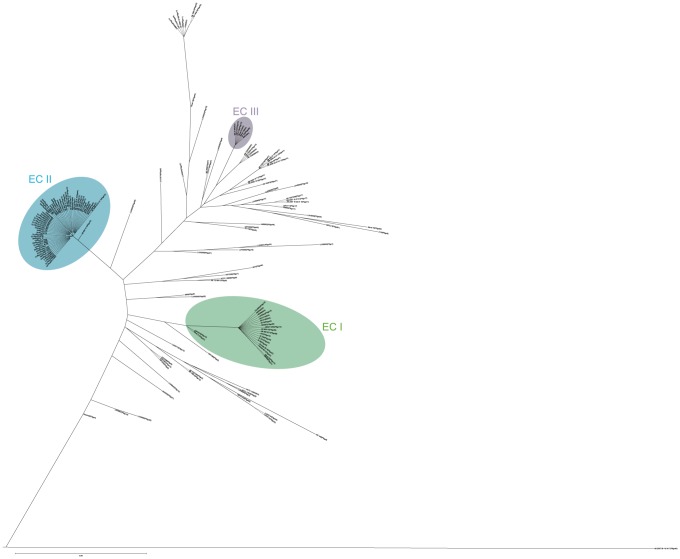
Tree for *A. baumannii* stains. Maximum likelihood tree using 6 house-keeping genes (*cpn60*, *fusA*, *pyrG, recA*, *rplB*, *ompA*) in 217 *Acinetobacter baumannii* genomes. The three EC groups shown are based on MLST STs as discussed in the text. The sequences aligned by Clustalw v2.0. The tree is generated by phyML v3.0 with the JC69 substitution model and 1000 bootstrap values.

The EC I, EC II and EC III clones were located in the tree (see Materials and Methods, Fig. 7) [39]. Two of the 25 PSgc type strains of the current study were found to be in EC II (PSgc5 and PSgc9), and one (PSgc13) in EC I. None were in EC III.

The genome sequences add six new PSgc forms to EC II, bringing the total number of polysaccharides in EC II to 9 (PSgc1, 5, 9, 12, 40, 44, 47, 52, 56). Strains of five serovars are found in EC I (PSgc13, 27, 39, 41, 43). A PSgc13 strain is also present in EC III which includes 2 serovars: PSgc13 and PSgc59 [35].

## Conclusions

We have sequenced the genomes of type strains for the 27 serovars of *A. baumannii* for which the strains are available, and extracted the gene clusters for synthesis of the polysaccharide responsible for serotype specificity. Twenty-five distinct sequence forms were found, and all have the same overall organisation with shared genes *wza*, *wzb* and *wzc* in one orientation at one end, a set of generally serovar-specific genes in the middle followed by a set of genes for glucose-related reactions that are mostly not specifically related to serotype specificity. All but *pgm*, the last of these genes, are transcribed in the opposite direction to the first three genes.

We have allocated a number to each of the polysaccharide gene cluster forms (PSgc1–PSgc27), using the original serovar numbers. One reason for changing the nomenclature is that some of the gene cluster sequences did not fit the reported structures and this could well be due to errors in strain maintenance or transfer between labs, and as the serotyping scheme was not widely used there is alternative strain collection for confirmation. A second reason is that the variation in the PSgc-specific genes enabled us to establish a molecular typing scheme based on variation in the major surface polysaccharide that was used previously by Traub to develop the serotyping scheme, and the new sequence forms have not been subjected to any serological analysis. We examined 190 *Acinetobacter* genome sequences. The gene cluster sequence could be determined for 165 of the genome sequences, and as the remaining 15 were not full genome sequences, the gene cluster may well have been present. We now have 77 distinct PSgc sequences in *Acinetobacter*. There are 5 of them in EC I, 9 in EC II and 2 in EC III.

The sequence diversity can be used to determine the PSgc form by a PCR-based test, or by using a microarray test as has been demonstrated for *Shigella* [63]. However *A. baumannii* isolates are often from one of the major clones, and serovar diversity within these clones is quite low. Molecular serotyping is more likely to be useful for determining the patterns of diversity outside of the major clones, or for screening large numbers of isolates for overall diversity, than in routine clinical screening.

The number of PSgc sequence forms is quite remarkable and we believe it reflects the level of antigenic diversity in *Acinetobacter*. Particularly interesting is the great diversity within EC II, which seems to have expanded worldwide.

## Supporting Information

Text S1 **The sugar pathway genes present in the 25 PSgc sequence forms.**
(DOC)

Text S2 **The** ***galU*** **,** ***ugd*** **,** ***pgi*** **,** ***gne*** **,** ***cgmA*** **and** ***pgm*** **genes, generally present in the** ***Acinetobacter*** **PSgc.**
(DOC)

Table S1 **The** ***Acinetobacter*** **strains used in this study.**
(DOC)

Table S2 **Characteristics of sugar-pathway genes in the** ***Acinetobacter*** **polysaccharides gene clusters for the 25 PSgc sequence forms.**
(DOC)

Table S3 **The** ***wzx*** **and** ***wzy*** **forms and unique genes in each PSgc.**
(DOC)

Table S4 A. Glycosyltransferase genes in the *Acinetobacter* polysaccharides gene clusters for the 25 PSgc sequence forms. B. Glycosyltransferase genes found in each gene cluster. C. Diversity of shared glycosyltransferase genes.
(DOC)

Table S5 ***wzy*** **genes in the** ***Acinetobacter*** **polysaccharides gene clusters for the 25 PSgc sequence forms.**
(DOC)

Table S6 **Primers used for** ***wzy*** **in** ***Acinetobacter*** **molecular typing.**
(DOC)

Table S7 **PSgc allocation of** ***Acinetobacter*** **strains with genome sequences.**
(DOC)
