# Supplementary material for: Diversity in the Major Polysaccharide Antigen of Acinetobacter Baumannii Assessed by DNA Sequencing, and Development of a Molecular Serotyping Scheme
Source: PLoS One. 2013 Jul 29;8(7):e70329. doi: 10.1371/journal.pone.0070329 (PMC3726653; doi:10.1371/journal.pone.0070329)
Supplement: Text S1 — The sugar pathway genes present in the 25 PSgc sequence forms. (DOC) [file pone.0070329.s001.doc]

**Text S1.**

**The sugar pathway genes present in the 25 PSgc sequence forms**

The genes are shown as part of the gene clusters in Figs 3 and 6, and the pathways are shown in Fig 4. The best-hit BLAST data is shown in Table S2.

In this section we summarise the evidence for the allocation of these genes to the specific pathways.

Synthesis of dTDP-D-Fuc3N(R3Hb) :

There is a known 2-gene pathway (*fdtA* and *fdtB*) for synthesis of dTDP-D-Fuc3N [1]. The genes *fdtA* and *fdtB* can be found in PSgc2 and PSgc25 by BLAST. For instance, *fdtA* and *fdtB* in PSgc2 share 49% and 58% identity in amino acid level to each homolog in *E. coli* O114, respectively (Table S2). Recently, FdhC has been identified in *E. coli* O103 as the dTDP-D-Fuc3N 3-hydroxybutanoyltransferase which is responsible for the synthesis of dTDP-D-Fuc3N(R3Hb) from dTDP-D-Fuc3N [2]. A putative butyryltransferase gene can be found in PSgc2, and its product shares 45% identity to FdhC in *E. coli* O103(Table S2).

Synthesis of UDP-L-FucNAc

FnlA, FnlB and FnlC have been identified to be responsible for the synthesis of UDP-L-FucNAc [3, 4]. *fnlABC* genes have been found to be involved in the synthesis of O-polysaccharides in several species. The *fnlABC* genes in PSgc5 and PSgc24 can be identified on the basis of their similarity to each homolog (57-83% identity in amino acid level) within the O-polysaccharide gene clusters of *E. coli* O145 (Table S2).

Synthesis of UDP-GalNAcA

This pathway has been studied in *Pseudomonas aeruginosa* O6, and has been revised recently [5]. The pathway includes two steps: oxygenation of UDP-GlcNAc to UDP-GlcNAcA followed by epimerisation to UDP-GalNAcA. The genes in *P. aeruginosa* are named *wbpO* and *wbpP*, respectively, and also have generic names which are now *gnaA* and *gnaB* respectively (manuscript in discussion with PLoS one).They can be found in several *Acinetobacter* polysaccharide gene clusters by BLAST in our study (Table S2).

Synthesis of UDP-GalNAc:

The synthesis of UDP-GalNAc from UDP-GlcNAc requires an epimerase encoded by *gne* [6], which is present in most *Acinetobacter* polysaccharide gene clusters and discussed in the main text.

Synthesis of CMP-Leg5Ac7Ac:

The pathway of CMP-Leg5Ac7Ac has been described [7], and seven genes (*lea1*-*lea7*) responsible for its derivative (CMP-Leg5Ac7Ala) have also been found in the gene clusters for the O-polysaccharide of *E. coli* O161 [8]. There are genes in PSgc1 and PSgc24 with good homology to 6 of the 7 CMP-Leg5Ac7Ala pathway genes of *E. coli* O161 (38-73% identity in amino acid level) (Table S2). The only exception is *lea3* which encodes an alanyltransferase. Furthermore, a putative acetyltransferase gene can be found among the homologs of CMP-Leg5Ac7Ala pathway genes in PSgc1 and PSgc24 (Table S2). This replacement may contribute the structural difference between CMP-Leg5Ac7Ac and CMP-Leg5Ac7Ala. Surprisingly there is only low level similarity of the *Acinetobacter* genes to those described by Watson et al [7]. In this study, we name 7 CMP-Leg5Ac7Ac pathway genes *leg1*-*7*, pending confirmation of function when they would be given traditional *leg*A-F names in function order.

Synthesis of UDP-ManNAc :

UDP-ManNAc is synthesized from UDP-GlcNAc by a UDP-N-acetylglucosamine 2-epimerase (MnaA) [9]. *mnaA* can be found in PSgc10 and PSgc18 by BLAST. For example, *mnaA* in PSgc10 share 62% identity in amino acid level to its homolog in *Shigella dysenteriae* type 10 (Table S2).

Synthesis of CMP-Pse5Ac7(R3Hb) :

The pathway for CMP-Pse5Ac7(R3Hb) has been found in *Shigella boydii* type 7, and includes 6 enzymes (Psb1-Psb6) [3, 4]. A set of *psb* genes, with the same order as the gene set in *Shigella boydii* type 7, can be found in PSgc12, PSgc25 and PSgc26 by BLAST. For instance, Psb1-6 in PSgc12 share 30-81% identity to their *Shigella boydii* type 7 homologs (Table S2).

Synthesis of dTDP-D-Qui3N(R3Hb) :

The situation for the synthesis of dTDP-D-Qui3N(R3Hb) is like the synthesis of dTDP-D-Fuc3N(R3Hb) in that the pathway for the basic sugar, Qui3N, is known but there is an additional butyryl moiety.

The biosynthesis of dTDP-d-Qui*p*3N involves QdtA and QdtB [10]. *qdtA* and *qdtB* can be found in PSgc23 by Blast, which share 63% and 68% identity (amino acid level) to their homologs involved in the synthesis of O-polysaccharide of *Providencia alcalifaciens* O40 (Table S2). A putative butyryltransferase gene is also found in PSgc23, and its product shares 50% identity to FdhC, which is a dTDP-D-Fuc3N 3-hydroxybutanoyltransferase[2]. We propose that this gene is responsible for the synthesis of dTDP-D-Qui3N(R3Hb) from dTDP-d-Qui*p*3N, and name it *qdh*C.

Synthesis of dTDP-L-Rha :

Rha is widely present in the bacterial surface polysaccharide, and its biosynthesis pathway is well known to involve 4 enzymes (RmlABCD)[3]. *rmlBDAC* are found in 4 *Acinetobacter* polysaccharide gene clusters by BLAST (Table S2) with the same gene order as that in the polysaccharide gene clusters of *E. coli*, *Shigella* and *Salmonella*.

Synthesis of UDP-GlcA :

UDP-GlcA is synthesized from UDP-Glc by UDP-Glc-6-dehydrogenase [11], and *ugd* is present in all *Acinetobacter* polysaccharide gene clusters (see main text and Text S2).

Synthesis of UDP-GlcNAc(3NAc)A :

This pathway has been discussed in the main text.

**References**

1. Pfoestl A, Hofinger A, Kosma P, Messner P (2003) Biosynthesis of dTDP-3-acetamido-3,6-dideoxy-alpha-D-galactose in *Aneurinibacillus thermoaerophilus* L420-91T. J Biol Chem 278: 26410-26417.

2. Liu B, Perepelov AV, Svensson MV, Shevelev SD, Guo D, et al. (2010) Genetic and structural relationships of *Salmonella* O55 and *Escherichia coli* O103 O-antigens and identification of a 3-hydroxybutanoyltransferase gene involved in the synthesis of a Fuc3N derivative. Glycobiology 20: 679-688.

3. Liu B, Knirel YA, Feng L, Perepelov AV, Senchenkova SN, et al. (2008) Structure and genetics of *Shigella* O antigens. FEMS Microbiol Rev 32: 627-653.

4. Kneidinger B, O'Riordan K, Li J, Brisson J, Lee J, et al. (2003) Three highly conserved proteins catalyze the conversion of UDP-N-acetyl-D-glucosamine to precursors for the biosynthesis of O antigen in *Pseudomonas aeruginosa* O11 and capsule in *Staphylococcus aureus* type 5. Implications for the UDP-N-acetyl-L-fucosamine biosynthetic pathway. J Biol Chem 278: 3615-3627.

5. Miller WL, Matewish MJ, McNally DJ, Ishiyama N, Anderson EM, et al. (2008) Flagellin glycosylation in *Pseudomonas aeruginosa* PAK requires the O-antigen biosynthesis enzyme WbpO. J Biol Chem 283: 3507-3518.

6. Bengoechea JA, Pinta E, Salminen T, Oertelt C, Holst O, et al. (2002) Functional characterization of Gne (UDP-N-acetylglucosamine-4-epimerase), Wzz (chain length determinant), and Wzy (O-antigen polymerase) of *Yersinia enterocolitica* serotype O:8. J Bacteriol 184: 4277-4287.

7. Watson DC, Leclerc S, Wakarchuk WW, Young NM (2011) Enzymatic synthesis and properties of glycoconjugates with legionaminic acid as a replacement for neuraminic acid. Glycobiology 21: 99-108.

8. Li X, Perepelov AV, Wang Q, Senchenkova SN, Liu B, et al. (2010) Structural and genetic characterization of the O-antigen of *Escherichia coli* O161 containing a derivative of a higher acidic diamino sugar, legionaminic acid. Carbohydr Res 345: 1581-1587.

9. Campbell RE, Mosimann SC, Tanner ME, Strynadka NC (2000) The structure of UDP-N-acetylglucosamine 2-epimerase reveals homology to phosphoglycosyl transferases. Biochemistry 39: 14993-15001.

10. Pfostl A, Zayni S, Hofinger A, Kosma P, Schaffer C, et al. (2008) Biosynthesis of dTDP-3-acetamido-3,6-dideoxy-alpha-D-glucose. Biochem J 410: 187-194.

11. Stevenson G, Andrianopoulos K, Hobbs M, Reeves P R (1996) Organization of the *Escherichia coli* K-12 gene cluster responsible for production of the extracellular polysaccharide colanic acid. J Bacteriol 178: 4885-4893.
