# Supplementary material for: Diversity in the Major Polysaccharide Antigen of Acinetobacter Baumannii Assessed by DNA Sequencing, and Development of a Molecular Serotyping Scheme
Source: PLoS One. 2013 Jul 29;8(7):e70329. doi: 10.1371/journal.pone.0070329 (PMC3726653; doi:10.1371/journal.pone.0070329)
Supplement: Text S2 — The galU , ugd , pgi , gne , cgmA and pgm genes, generally present in the Acinetobacter PSgc. (DOC) [file pone.0070329.s002.doc]

**Text S2.**

**The *galU*, *ugd*, *pgi*, *gne*, *cgmA* and *pgm* genes*,* generally present in the *Acinetobacter* PSgc**

The *galU*, *ugd*, *pgi*, *gne*, *cgmA* and *pgm* are present in all or most of the *Acinetobacter* PSgc. Some are required for synthesis of some the polysaccharide structure, but in other species these genes are usually not in the PSgc, but elsewhere on the chromosome, as they have a wider role in metabolism. They were discussed very briefly in the main text and some further information is given below.

*galU* is required for synthesis of UDP-glucose and in *E. coli* this is a central metabolism gene as it is required for entry to the pentose and gluconate pathways. However the KEGG pathway charts (<http://www.genome.jp/kegg-bin/show_organism?org=acb>) show that neither pathway is present in *Acinetobacter* so UDP-glucose appears to be used only for polysaccharide synthesis, where it is the starting point for many sugar biosynthesis pathways. This may account for it being located with other genes for sugar biosynthesis.

*ugd* codes for UDP-glucose 6-dehydrogenase required for synthesis of UDP-GlcA, not present in our structures but is found in other *Acinetobacter* polysaccharide structures [25]. UGD is also required for entry into the pentose phosphate pathway discussed above.

*pgm* codes for phosphoglucomutase (PGM) that interconverts glucose-1-phosphate and glucose-6-phosphate. However BLAST searches show that the *Acinetobacter* protein is in a group with bifunctional PMM/PGM products that work with both mannose and glucose derivatives [50]. The *Pseudomonas* PGM gene [51] is also in this group and acts in synthesis of GDP-mannose derivatives as above but also on glucose phosphates where it is required for entry of exogenous glucose into glycolysis [52], whereas in the *Enterobacteriacea* there is characteristically a monofunctional *pgm* gene for the glucose reaction and a *manB* gene coding for a PMM enzyme involved in GDP-mannose synthesis. The bifunctional PMM/PGM proteins in their PMM role are required for synthesis of mannose, and also sugars such as L-fucose, with pathways that start with GDP-mannose. None of these sugars are in any of our structures, but are reported for O antigens of other *Acininetobacter* spp [25]. PMM genes are commonly known as *manB* genes and as a result this name has been given to the genes for bifunctional PMM/PGM proteins in many *A. baumannii* genome sequences.

*pgi* codes for phosphoglucose isomerase, involved in central metabolism by interconverting α-D-Glucose 6-phosphate and β-D-Fructose 6-phosphate, and is probably in all bacteria as it is involved in the Embden-Meyerhof pathway for glycolysis and also for gluconeogensis. The glycolysis pathway appears not to function as such in *Acinetobacter* as the pyruvite kinase gene for the final step is missing, and most *Acinetobacter* cannot grow on glucose [53]. The other steps are presumably used only for biosynthesis.

The *cgmA* gene is found in *Mesorhizobium loti* where it confers ability to add glycerol phosphate to cyclic b-1,2-glucans. There are no such substitutions in the reported *A. baumannii* polysaccharide structures and there is no obvious role for the gene in the 11 sequences for which we have a structure. In *M. loti* these glucans are present in the periplasm, where they are thought to act as osmoprotectants by contributing to the maintenance of osmolarity of the periplasm as shown for *E. coli* [56]. The role of *cgmA* in *A. bumannii* is not clear at present.

**References**

25. Knirel YA (2011) Structure of O-antigens. In: Valvano MA, Knirel YA, editors. Bacterial lipopolysaccharide: Springer Verlag. pp. 42-108.

50. Shackelford GS, Regni CA, Beamer LJ (2004) Evolutionary trace analysis of the alpha-D-phosphohexomutase superfamily. Protein Sci 13: 2130-2138.

51. Regni C, Naught L, Tipton PA, Beamer LJ (2004) Structural basis of diverse substrate recognition by the enzyme PMM/PGM from *P. aeruginosa*. Structure 12: 55-63.

52. Fraenkel (1996) Glycolysis. In: Neidhardt FC, Curtiss R, Ingraham JL, Lin ECC, Low KB et al., editors. *Escherichia and Salmonella* Cellular and Molecular Biology. Washington, D. C.: ASM Press. pp. 189-198.

53. Young DM, Parke D, Ornston LN (2005) Opportunities for genetic investigation afforded by *Acinetobacter baylyi*, a nutritionally versatile bacterial species that is highly competent for natural transformation. Annu Rev Microbiol 59: 519-551.

56. Kennedy EP (1996) Membrane-derived oligosaccharides

(periplasmic beta-D-glucans) of *Escherichia coli*. In: Neidhardt FC, Curtiss RI, Ingraham JL, Lin ECC, Low KB et al., editors. Escherichia

Coli and Salmonella Cellular and Molecular Biology, 2nd edn. Washington DC: ASMPress. pp. 1064–1071.
