# Supplementary material for: Diversity in the Major Polysaccharide Antigen of Acinetobacter Baumannii Assessed by DNA Sequencing, and Development of a Molecular Serotyping Scheme
Source: PLoS One. 2013 Jul 29;8(7):e70329. doi: 10.1371/journal.pone.0070329 (PMC3726653; doi:10.1371/journal.pone.0070329)
Supplement: Table S1 — The Acinetobacter strains used in this study. (DOC) [file pone.0070329.s003.doc]

**Table S1. The *Acinetobacter* strains used in this study**

| **Strain** | **Lab collection No.** | **Species** | **Original Serovar** | **Current Serovar** | **PSgc forms** | **Whole genome accession No.^a^** | **Gene cluster accession No.^b^** |
| --- | --- | --- | --- | --- | --- | --- | --- |
| LUH5533 | G4765 | baumannii | O1 | Sv1 | PSgc1 | DRS005660 | KC526894 |
| LUH3483 | G4760 | nosocomialis | O2 | Sv2 | PSgc2 | DRS005640 | KC526907 |
| LUH5534 | G4766 | baumannii | O3 | Sv3 | PSgc3 | DRS005641 | KC526908 |
| LUH5536 | G4768 | nosocomialis | O4 | Sv4 | PSgc4 | DRS005652 | KC526912 |
| LUH3484 | G4761 | baumannii | O5 | Sv5 | PSgc5 | DRS005649 | KC526895 |
| LUH5535 | G4767 | baumannii | O6 | Sv6 | PSgc6 | DRS005643 | KC526896 |
| LUH5537 | G4769 | baumannii | O7 | Sv9 | PSgc9 | DRS005639 | KC526920 |
| LUH5538 | G4770 | baumannii | O8 | Sv8 | PSgc8 | DRS005645 | KC526898 |
| LUH5539 | G4771 | baumannii | O9 | Sv9 | PSgc9 | DRS005655 | KC526915 |
| LUH5540 | G4772 | baumannii | O10 | Sv10 | PSgc10 | DRS005653 | KC526902 |
| LUH5541 | G4773 | nosocomialis | O11 | Sv11 | PSgc11 | DRS005637 | KC526906 |
| LUH3713 | G4763 | baumannii | O12 | Sv12 | PSgc12 | DRS005634 | KC526916 |
| LUH5542 | G4774 | baumannii | O13 | Sv13 | PSgc13 | DRS005654 | KC526901 |
| LUH5543 | G4775 | baumannii | O14 | Sv14 | PSgc14 | DRS005636 | KC526913 |
| LUH5544 | G4776 | baumannii | O15 | Sv15 | PSgc15 | DRS005646 | KC526905 |
| LUH3712 | G4762 | baumannii | O16 | Sv23 | PSgc23 | DRS005638 | KC526914 |
| LUH5545 | G4777 | baumannii | O17 | Sv17 | PSgc17 | DRS005658 | KC526904 |
| LUH5546 | G4778 | baumannii | O18 | Sv18 | PSgc18 | DRS005650 | KC526899 |
| LUH5547 | G4779 | baumannii | O19 | Sv19 | PSgc19 | DRS005644 | KC526918 |
| LUH5548 | G4780 | baumannii | O20 | Sv20 | PSgc20 | DRS005635 | KC526910 |
| LUH5549 | G4781 | baumannii | O21 | Sv21 | PSgc21 | DRS005651 | KC526897 |
| LUH5550 | G4782 | baumannii | O22 | Sv22 | PSgc22 | DRS005642 | KC526903 |
| LUH3714 | G4764 | baumannii | O23 | Sv23 | PSgc23 | DRS005656 | KC526911 |
| LUH5551 | G4783 | baumannii | O24 | Sv24 | PSgc24 | DRS005659 | KC526909 |
| LUH5552 | G4784 | baumannii | O25 | Sv25 | PSgc25 | DRS005657 | KC526919 |
| LUH5553 | G4785 | baumannii | O26 | Sv26 | PSgc26 | DRS005648 | KC526917 |
| LUH5554 | G4786 | baumannii | O27 | Sv27 | PSgc27 | DRS005647 | KC526900 |

1. The raw data of whole genomes were submitted to DDBJ (DNA Data Bank of Japan) database.
2. The gene-clusters sequences were submitted to NCBI (National Center for Biotechnology Information) database.
