# Supplementary material for: Diversity in the Major Polysaccharide Antigen of Acinetobacter Baumannii Assessed by DNA Sequencing, and Development of a Molecular Serotyping Scheme
Source: PLoS One. 2013 Jul 29;8(7):e70329. doi: 10.1371/journal.pone.0070329 (PMC3726653; doi:10.1371/journal.pone.0070329)
Supplement: Table S2 — Characteristics of sugar-pathway genes in the Acinetobacter polysaccharides gene clusters for the 25 PSgc sequence forms. (DOC) [file pone.0070329.s004.doc]

**Table S2. Characteristics of sugar-pathway genes in the *Acinetobacter* polysaccharides gene clusters for the 25 PSgc sequence forms**

| **Pathway** | **Gene Name** | **Putative function** | **PSgc form** | **GC%** | **Length of amino acids** | **Conserved domain^a^** | **Similar protein in Swiss-Prot database**  **function, OS=strain,GN=gene, (accession No.)** | **%Identical/%Similar**  **for Swiss-Prot database** | **Gene name, species , (strain), accession No. of the Similar protein** | **%Identical/%Similar for nr database** | **Present in other PSgc form** | **Diversity of shared gene (%Identical)** |
| --- | --- | --- | --- | --- | --- | --- | --- | --- | --- | --- | --- | --- |
| Synthesis of dTDP-D-Fuc3N(R3Hb) | *fdtA* | dTDP-6-deoxyhex-4-ulose isomerase | PSgc2 | 31.3 | 132 | FdtA, PF05523, E=3.1e-52 | TDP-4-oxo-6-deoxy-alpha-D-glucose-3,4-oxoisomerase OS=*Aneurinibacillus thermoaerophilus* GN=*fdtA*, (Q6T1W8|FDTA_ANETH) | 58/76 | FdtA, *E.coli* O114, (AAT77171) | 49/65 | PSgc25 | 71 |
|  | *fdtB* | dTDP-6-deoxy-D-*xylo*-hex-3-uloseaminase | PSgc2 | 35.3 | 372 | DegT_DnrJ_EryC1, PF01041, E=2e-116 | dTDP-3-amino-3,6-dideoxy-alpha-D-galactopyranose transaminase OS=*Aneurinibacillus thermoaerophilus* GN=*fdtB*, (Q6T1W6\|FDTB_ANETH) | 65/77 | FdtB, *E.coli* O114, (AAT77174) | 58/73 | PSgc25 | 96 |
|  | *fdhC* | butyryltransferase | PSgc2 | 27.3 | 180 | N/A | Butyryltransferase OS=*Vibrio harveyi* (strain HY01) GN=A1Q_0139, (A6ASH4\|A6ASH4_VIBHA) | 56/69 | butyryltransferase, *E.coli* O103, (AAS73166) | 45/64 | N/A | N/A |
| Synthesis of UDP-L-FucNAc | *fnlA* | 4,6-dehydratase,3-and 5-epimerizase | PSgc24 | 36.5 | 345 | Polysacc_synt_2, PF02719, E=2.7e-109 | UDP-N-acetylglucosamine 4,6-dehydratase OS= *Campylobacter jejuni* (strain 81-176) GN=*pseB,* (Q5QKR8|PSEB_CAMJJ) | 40/57 | FnlA, *E.coli* O177, (AAY28260) | 83/91 | PSgc5 | 99 |
|  | *fnlB* | reductase | PSgc24 | 37.7 | 369 | Epimerase, PF01370, E=6.8e-14 | NAD dependent epimerase/dehydratase family protein OS=*Acinetobacter baumannii* (strain OIFC098) GN=ACIN5098_0097, (K5PKF3\|K5PKF3_ACIBA) | 99/99 | FnlB, *E.coli* O145, (AAV74536) | 57/73 | PSgc5 | 99 |
|  | *fnlC* | C-2 epimerase | PSgc24 | 37.2 | 370 | Epimerase_2, PF02350, E=3.3e-91 | UDP-N-acetylglucosamine 2-epimerase OS=*Methanocaldococcus jannaschii* (strain ATCC 43067 / DSM 2661 / JAL-1 / JCM 10045 / NBRC 100440) GN=*wecB*, (Q58899\|WECB_METJA) | 32/58 | FnlC, *E.coli* O15, (AAV74546) | 71/84 | PSgc5 | 99 |
| Synthesis of UDP-GalNAcA | *gnaA* | UDP-glucose/GDP-mannose dehydrogenase | PSgc1 | 36.4 | 162 | UDPG_MGDP_dh_C, PF03720, E=9.3e-23 | Vi polysaccharide biosynthesis protein VipA/TviB OS=*Salmonella typhi* GN=*vipA*, (Q04972|VIPA_SALTI) | 69/87 | WbpO, *Pseudomonas aeruginosa*, (AAM27816) | 59/79 | All PSgc | 63 ~ 99 |
|  | *gnaB* | NAD-dependent epimerase/dehydratase | PSgc13 | 35.5 | 340 | Epimerase, PF01370, E=1.4e-54 | Vi polysaccharide biosynthesis protein VipB/TviC OS=*Salmonella typhi* GN=*vipB*, (Q04973\|VIPB_SALTI) | 69/80 | WbpP, *Pseudomonas aeruginosa*, (AAM27817) | 74/86 | PSgc17,19,5,6,8 | 26 ~ 99 |
| Synthesis of UDP-GalNAc | *gne* | UDP-GlcNAc 4-epimerase | PSgc1 | 38.8 | 338 | Epimerase, PF01370, E=5.1e-53 | UDP-glucose 4-epimerase OS=*Bacillus subtilis* (strain 168) GN=*galE*, (P55180|GALE_BACSU) | 63/76 | UDP-glucose 4-epimerase *Alteromonas macleodii* str. 'Deep ecotype', (YP_004426106) | 59/76 | PSgc11,12,15,18,2,20,21,22,23,24,25,26,27,3,4,5,6,9 | 79 ~ 100 |
| Synthesis of CMP-Leg5Ac7Ac | *leg1* | NAD-dependent epimerase/dehydratase | PSgc1 | 33.6 | 398 | Polysacc_synt_2, PF02719, E=9e-46 | UDP-N-acetyl-alpha-D-glucosamine C6 dehydratase OS=*Campylobacter jejuni* *subsp. jejuni* serotype O:2 (strain NCTC 11168) GN=*pglF*, (Q0P9D4|PGLF_CAMJE) | 26/44 | Lea1, *E.coli* O161, (ADJ19193) | 73/82 | PSgc24 | 100 |
|  | *leg2* | aminotransferase | PSgc1 | 35.1 | 382 | DegT_DnrJ_EryC1, PF01041, E=3.4e-79 | UDP-4-amino-4,6-dideoxy-N-acetyl-alpha-D-glucosamine transaminase OS=*Campylobacter jejuni subsp. jejuni* serotype O:2 (strain NCTC 11168) GN=*pglE*, (Q0P9D3\|PGLE_CAMJE) | 33/55 | Lea2, *E.coli* O161, (ADJ19194) | 62/80 | PSgc24 | 99 |
|  | *leg5* | NDP-sugar hydrolase/epimerase | PSgc1 | 36.6 | 378 | Epimerase_2, PF02350, E=3e-92 | 2,3,4,5-tetrahydropyridine-2,6-dicarboxylate N-acetyltransferase OS=*Thermosipho africanus* (strain TCF52B) GN=*dapH*, (B7IF15\|DAPH_THEAB) | 34/51 | Lea5, *E.coli* O161, (ADJ19197) | 47/64 | PSgc24 | 99 |
|  | *leg4* | legionaminic acid synthase | PSgc1 | 34.4 | 364 | NeuB, PF03102, E=4.5e-86 | Pseudaminic acid synthase OS= *Campylobacter jejuni* (strain NCTC 11168) GN= *pseI*, (Q0P8U0\|PSEI_CAMJE) | 30/51 | Lea4, *E.coli* O161, (ADJ19196) | 38/57 | PSgc24 | 64 |
|  | *leg3* | acetyltransferase | PSgc1 | 37.2 | 213 | N/A | Polysialic acid biosynthesis protein P7 OS=*Escherichia coli* GN=*neuC*, (Q47400\|NEUC_ECOLX) | 37/58 | acetyltransferase, *Fusobacterium ulcerans*, (ZP_10974326) | 31/53 | PSgc24 | 53 |
|  | *leg6* | nucleotidase | PSgc1 | 32.9 | 344 | NTP_transferase, PF00483, E=1.5e-35 | Probable mannose-1-phosphate guanylyltransferase 1 OS=*Oryza sativa subsp. japonica* GN=Os03g0268400, (Q84JH5\|GMPP1_ORYSJ) | 36/54 | Lea6, *E.coli* O161, (ADJ19199) | 51/73 | PSgc24 | 95 |
|  | *leg7* | cytidylyltransferase | PSgc1 | 35.3 | 235 | CTP_transf_3, PF02348, E=1.2e-29 | Probable N-acylneuraminate cytidylyltransferase OS=*Haemophilus influenzae* (strain ATCC 51907 / DSM 11121 / KW20 / Rd) GN=*neuA*, (Q57140\|NEUA_HAEIN) | 35/54 | Lea7, *E.coli* O161, (ADJ19200) | 45/66 | PSgc24 | 92 |
| Synthesis of UDP-ManNAc | *mnaA* | UDP-N-acetylglucosamine 2-epimerase | PSgc10 | 31.2 | 378 | Epimerase_2, PF02350, E=2.4e-125 | UDP-N-acetylglucosamine 2-epimerase OS=*Yersinia pestis* GN=*wecB*, (Q8ZAE3|WECB_YERPE) | 63/78 | MnaA, *Shigella dysenteriae* type 10, (ACA24905) | 62/79 | PSgc18 | 64 |
| Synthesis of CMP-Pse5Ac7(R3Hb) | *psb1* | C6 dehydratase/C5 epimerase | PSgc12 | 36.2 | 332 | Polysacc_synt_2, PF02719, E=1.3e-96 | UDP-N-acetylglucosamine 4,6-dehydratase (inverting) OS=*Campylobacter jejuni* subsp*. jejuni* serotype O:23/36 (strain 81-176) GN=*pseB*, (Q5QKR8|PSEB_CAMJJ) | 58/73 | Psb1, *Shigella boydii* type 7, (ACD37064) | 81/90 | PSgc22 PSgc26 | 99 |
|  | *psb2* | aminotransferase | PSgc12 | 36.3 | 386 | DegT_DnrJ_EryC1, PF01041, E=3.1e-105 | UDP-4-amino-4-deoxy-L-arabinose--oxoglutarate aminotransferase OS=*Proteus mirabilis* (strain HI4320) GN=*arnB*, (B4ETL5\|ARNB_PROMH) | 34/56 | Psb2, *S. boydii* type 7, (ACD37065) | 64/79 | PSgc22 PSgc26 | 99 |
|  | *psb3* | cytidylyltransferase | PSgc12 | 42.4 | 230 | CTP_transf_3, PF02348, E=3.1e-38 | Pseudaminic acid cytidylyltransferase OS=*Campylobacter jejuni subsp. jejuni* serotype O:2 (strain NCTC 11168) GN=*pseF*, (Q0P8U6\|PSEF_CAMJE) | 52/70 | Psb3, *S. boydii* type 7, (ACD37066) | 56/74 | PSgc22 PSgc26 | 91 ~ 99 |
|  | *psb4* | nucleotidase | PSgc12 | 39.0 | 365 | Glyco_tran_28_C, PF04101, E=1.6e-5 | UDP-2,4-diacetamido-2,4,6-trideoxy-beta-L-altropyranose hydrolase OS=*Campylobacter jejuni subsp. jejuni* serotype O:2 (strain NCTC 11168) GN=*pseG*, (Q0P8U5\|PSEG_CAMJE) | 29/45 | Psb4, *S. boydii* type 7, (ACD37067) | 38/59 | PSgc22 PSgc26 | 32 ~ 99 |
|  | *psb5* | N-(3-hydroxybutanoyl) transferase | PSgc12 | 35.9 | 171 | Acetyltransf_1, PF00583, E=3.4e-5 | Acetyltransferase PseH OS=*Campylobacter jejuni subsp. jejuni* serotype O:23/36 (strain 81-176) GN=*pseH*, (A1W0U7\|PSEH_CAMJJ) | 29/55 | Psb5, *S. boydii* type 7, (ACD37068) | 30/47 | PSgc22 PSgc26 | 34 ~ 99 |
|  | *psb6* | condensase | PSgc12 | 39.8 | 349 | NeuB, PF03102, E=1.2e-93 | Pseudaminic acid synthase OS=*Helicobacter pylori* (strain ATCC 700392 / 26695) GN=*pseI*, (O24980\|PSEI_HELPY) | 46/65 | Psb6, *S.boydii* type 7], (ACD37069) | 74/83 | PSgc22 PSgc26 | 91 ~ 99 |
| Synthesis of dTDP-D-Qui3N(R3Hb) | *qdtA* | dTDP-6-deoxy-3,4-keto-hexulose isomerase | PSgc23 | 33.3 | 127 | FdtA, PF05523, E=8.8e-49 | TDP-4-oxo-6-deoxy-alpha-D-glucose-3,4-oxoisomerase OS=*Aneurinibacillus thermoaerophilus* GN=*fdtA*, (Q6T1W8|FDTA_ANETH) | 58/76 | QdtA, *Providencia alcalifaciens* O40, (AEB61512) | 63/80 | N/A | N/A |
|  | *qdtB* | aminotransferase | PSgc23 | 35.5 | 371 | DegT_DnrJ_EryC1, PF01041, E=5.2e-118 | dTDP-3-amino-3,6-dideoxy-alpha-D-galactopyranose transaminase OS=Aneurinibacillus thermoaerophilus GN=*fdtB*, (Q6T1W6\|FDTB_ANETH) | 64/76 | QdtB, *P alcalifaciens* O40, (AEB61513) | 68/80 | N/A | N/A |
|  | *qdhC* | butyryltransferase | PSgc23 | 29.6 | 179 | N/A | Putative butyryltransferase OS=Cronobacter sakazakii (strain 696) GN=BN128_1580, (K8CQN3\|K8CQN3_CROSK) | 49/69 | butyryltransferase, *E.coli* O103, (AAS73166) | 50/64 | N/A | N/A |
| Synthesis of dTDP-L-Rha | *rmlA* | glucose-1-phosphate thymidylyltransferase | PSgc10 | 34.6 | 297 | NTP_transferase, PF00483, E=7e-70 | Glucose-1-phosphate thymidylyltransferase OS=*Salmonella typhimurium* (strain LT2 / SGSC1412 / ATCC 700720) GN=*rmlA*, (P26393|RMLA_SALTY) | 66/82 | RmlA, *E.coli* O109, (ADR74236) | 70/82 | PSgc14,17,23,19,2,25,8 | 70 ~ 99 |
|  | *rmlB* | dTDP-D-glucose-4,6-dehydratase | PSgc10 | 35.4 | 358 | Epimerase, PF01370, E=9.3e-78 | dTDP-glucose 4,6-dehydratase 2 OS=*Escherichia coli* (strain K12) GN=*rffG*, (P27830\|RMLB2_ECOLI) | 75/86 | dTDP-glucose 4,6-dehydratase, *E.coli* 536, (YP_671844) | 75/85 | PSgc14,17,19,2,23,25,8 | 73 ~ 99 |
|  | *rmlC* | dTDP-4-keto-6-deoxy-D-glucose-3,5-epimerase | PSgc10 | 33.3 | 188 | dTDP_sugar_isom, PF00908, E=2.6e-74 | dTDP-4-dehydrorhamnose 3,5-epimerase OS=*Shigella flexneri* GN=*rfbC*, (P37780\|RMLC_SHIFL) | 63/75 | RmlC, *E.coli* O147, (ABI98984) | 62/76 | PSgc14,17,19,8 | 73 ~ 99 |
|  | *rmlD* | dTDP-6-deoxy-L-mannose dehydrogenase | PSgc10 | 36.1 | 297 | RmlD_sub_bind, PF04321, E=2.2e-98 | dTDP-4-dehydrorhamnose reductase OS=*Escherichia coli* (strain K12) GN=*rfbD*, (P37760\|RMLD_ECOLI) | 52/69 | RmlD, *E.coli* O147, (AAY28253) | 51/69 | PSgc14, 17,19,8 | 56 ~ 99 |
| Synthesis of UDP-GlcA | *ugd* | UDP-glucose 6-dehydrogenase | PSgc1 | 37.7 | 420 | UDPG_MGDP_dh_C, PF03720, E=1.5e-25 | UDP-glucose 6-dehydrogenase OS=*Pseudomonas aeruginosa* (strain ATCC 15692 / PAO1 / 1C / PRS 101 / LMG 12228) GN=*udg*, (O86422|UDG_PSEAE) | 32/51 | UDP-glucose 6-dehydrogenase, *Halomonas* *sp.* TD01, (ZP_08638223) | 46/62 | All PSgc | 23 ~ 100 |
| Synthesis of UDP-GlcNAc(3NAc)A | *mnnA* | UDP-D-GlcNAcA oxidase | PSgc27 | 36.2 | 316 | GFO_IDH_MocA, PF01408, E=6e-22 | UDP-N-acetyl-2-amino-2-deoxy-D-glucuronate oxidase OS=*Pseudomonas aeruginosa* (strain ATCC 15692 / PAO1 / 1C / PRS 101 / LMG 12228) GN=*wbpB*, (G3XD23|WBPB_PSEAE) | 60/75 | WbpB, *Pseudomonas aeruginosa* O5, (AAC45853) | 59/74 | PSgc9 | 99 |
|  | *mnnC* | acyltransferase | PSgc27 | 42.5 | 192 | N/A | UDP-2-acetamido-3-amino-2,3-dideoxy-D-glucuronate N-acetyltransferase OS=*Pseudomonas aeruginosa* (strain ATCC 15692 / PAO1 / 1C / PRS 101 / LMG 12228) GN=*wbpD*, (G3XD01\|WBPD_PSEAE) | 83/92 | WbpD, *P. aeruginosa* O5, (AAG06544) | 82/92 | PSgc9 | 98 |
|  | *mnnB* | glutamine--scyllo-inositol transaminase | PSgc27 | 38.6 | 359 | DegT_DnrJ_EryC1, PF01041, E=1e-118 | UDP-2-acetamido-2-deoxy-3-oxo-D-glucuronate aminotransferase OS=*Pseudomonas aeruginosa* (strain ATCC 15692 / PAO1 / 1C / PRS 101 / LMG 12228) GN=*wbpE*, (Q9HZ76\|WBPE_PSEAE) | 79/88 | WbpE, *P. aeruginosa* O5, (AAG06543) | 78/88 | PSgc9 | 95 |
| Not known | *wbpL* | UDP-N-acetylmuramyl pentapeptide phosphotransferase/UDP-N-acetylglucosamine-1-phosphate transferase | PSgc24 | 30.0 | 336 | Glycos_transf_4, PF00953, E=3.4e-32 | Probable undecaprenyl-phosphate N-acetylglucosaminyl 1-phosphate transferase OS=*Bacillus subtilis* (strain 168) GN=*tagO*, (O34753|TAGO_BACSU) | 32/52 | glycosyltransferase, *P. aeruginosa* O5, (AAG06533) | 49/66 | PSgc5 | 99 |
| Not known | *wbpV* | function unknown | PSgc24 | 31.6 | 311 | Epimerase, PF01370, E=1.1e-30 | UDP-glucose 4-epimerase OS=*Vibrio cholerae* GN=*galE*, (Q56623|GALE_VIBCL) | 46/62 | WbpV, *P. aeruginosa* O6, (AAF23991) | 43/63 | PSgc5 | 99 |
| Initial transferase | *weeH* | initial transferase | PSgc1 | 38.7 | 155 | Bac_transf, PF02397, E=1.9e-48 | Undecaprenyl-phosphate galactose phosphotransferase OS=*Salmonella typhimurium* (strain LT2/ SGSC1412 / ATCC 700720) GN=*rfbP,* (P26406|RFBP_SALTY) | 54/71 | undecaprenyl-phosphate galactose phosphotransferase, *P. aeruginosa* DK2, (P_006481715) | 60/77 | All PSgc | 59 ~ 100 |
| Not known | *weeI* | acetyltransferase | PSgc13 | 37.6 | 216 | N/A | Putative acetyltransferase EpsM OS=*Bacillus subtilis* (strain 168) GN=*epsM*, (P71063|EPSM_BACSU) | 32/53 | acetyl transferase, *Pseudomonas sp.* Ag1, (ZP_10478394) | 56/73 | PSgc27 PSgc6 | 65 ~ 91 |
| Not known | *weeJ* | aminotransferase | PSgc13 | 38.7 | 391 | DegT_DnrJ_EryC1, PF01041, E=1.3e-97 | UDP-4-amino-4-deoxy-L-arabinose--oxoglutarate aminotransferase OS=*Pseudomonas fluorescens* (strain Pf0-1) GN=*arnB*, (Q3KCC3|ARNB_PSEPF) | 36/54 | aminotransferase, *Comamonas testosteroni* KF-1, (ZP_03541148) | 69/81 | PSgc27 PSgc6 | 97 ~ 98 |
| Not known | *weeK* | dehydratase/epimerase | PSgc13 | 37.1 | 605 | Polysacc_synt_2, PF02719, E=3.2e-127 | Capsular polysaccharide biosynthesis protein CapD OS=*Staphylococcus aureus* GN=*capD*, (P39853|CAPD_STAAU) | 43/64 | WbpM, *P. aeruginosa* O5, (NP_251831) | 44/62 | PSgc27 PSgc6 | 98 ~ 99 |
| Not known | *cgmA* | cyclic beta-1,2-glucan modification transmembrane protein | PSgc10 | 32.1 | 586 | Sulfatase, PF00884, E=1.4e-41 | Putative cyclic beta-1,2-glucan modification protein OS=*Rhizobium meliloti* (strain 1021) GN=*cgmA*, (P72302|CGMA_RHIME) | 34/51 | sulfatase, *Sinorhizobium medicae* WSM419, (YP_001327243) | 28/46 | PSgc11,12,14,15,17,18 ,19,2,20,23,25,3,4,8,9 | 88 ~ 100 |
|  | *galU* | UTP-glucose-1-phosphate uridylyltransferase | PSgc1 | 39.2 | 291 | NTP_transferase, PF00483, E=3.3e-22 | UTP--glucose-1-phosphate uridylyltransferase OS=*Haemophilus influenzae* (strain ATCC 51907 / DSM 11121 / KW20 / Rd) GN=*galU*, (P44878|GALU_HAEIN) | 57/73 | UTP-glucose-1-phosphate uridylyltransferase, *E.coli* PCN033, (EGP25421) | 55/72 | All PSgc | 79 ~ 100 |
|  | *gpi* | glucose-6-phosphate isomerase | PSgc1 | 37.6 | 556 | PGI, PF00342, E=5.6e-164 | Glucose-6-phosphate isomerase OS=*Acinetobacter baumannii* (strain ATCC 17978 / NCDC KC 755) GN=*pgi*, (A3M0W5|G6PI_ACIBT) | 99/99 | glucose-6-phosphate isomerase, *E.coli*, (YP_006093861) | 48/65 | All PSgc | 86 ~ 99 |
|  | *pgm* | phosphoglucomutase | PSgc1 | 40.8 | 456 | PGM_PMM_I, PF02878, E=7.1e-34 | Phosphomannomutase OS=*Vibrio cholerae* serotype O1 (strain ATCC 39315 / El Tor Inaba N16961) GN=*rfbB*, (Q06951|RFBB_VIBCH) | 64/78 | phosphomannomutase, *Vibrio cholerae* O395, (ACP08299) | 64/78 | All PSgc | 96 ~ 100 |

a, Pfam family (http://pfam.janelia.org/). “N/A” indicates there isn’t a Pfam family fit the gene.
