# Supplementary material for: Diversity in the Major Polysaccharide Antigen of Acinetobacter Baumannii Assessed by DNA Sequencing, and Development of a Molecular Serotyping Scheme
Source: PLoS One. 2013 Jul 29;8(7):e70329. doi: 10.1371/journal.pone.0070329 (PMC3726653; doi:10.1371/journal.pone.0070329)
Supplement: Table S3 — The wzx and wzy forms and unique genes in each PSgc. (DOC) [file pone.0070329.s005.doc]

**Table S3. The *wzx* and *wzy* forms and unique genes in each PSgc**

| **PSgc form** | ***wzx*** | ***wzy*** | **Number of unique genes** |
| --- | --- | --- | --- |
| PSgc1 | *wzx_1* | *wzy_1* | 1 |
| PSgc2 | *wzx_2* | *wzy_2* | 4 |
| PSgc3 | *wzx_3* | *wzy_3* | 5 |
| PSgc4 | *wzx_4* | *wzy_4* | 2 |
| PSgc5 | *wzx_5* | *wzy_5* | 4 |
| PSgc6 | *wzx_6* | *wzy_6* | 5 |
| PSgc8 | *wzx_7* | *wzy_7* | 2 |
| PSgc9 | *wzx_8* | *wzy_8* | 5 |
| PSgc10 | *wzx_9* | *wzy_9* | 3 |
| PSgc11 | *wzx_4* | *wzy_10* | 1 |
| PSgc12 | *wzx_10* | *wzy_11* | 4 |
| PSgc13 | *wzx_11* | *wzy_12* | 4 |
| PSgc14 | *wzx_12* | *wzy_13* | 3 |
| PSgc15 | *wzx_13* | *wzy_14* | 3 |
| PSgc17 | *wzx_7* | *wzy_15* | 2 |
| PSgc18 | *wzx_14* | *wzy_16* | 4 |
| PSgc19 | *wzx_15* | *wzy_17* | 5 |
| PSgc20 | *wzx_16* | *wzy_18* | 4 |
| PSgc21 | *wzx_17* | *wzy_19* | 7 |
| PSgc22 | *wzx_18* | *wzy_20* | 2 |
| PSgc23 | *wzx_2* | *wzy_21* | 7 |
| PSgc24 | *wzx_19* | *wzy_22* | 4 |
| PSgc25 | *wzx_2* | *wzy_23* | 4 |
| PSgc26 | *wzx_1* | *wzy_24* | 1 |
| PSgc27 | *wzx_20* | *wzy_25* | 5 |
