# Supplementary material for: Diversity in the Major Polysaccharide Antigen of Acinetobacter Baumannii Assessed by DNA Sequencing, and Development of a Molecular Serotyping Scheme
Source: PLoS One. 2013 Jul 29;8(7):e70329. doi: 10.1371/journal.pone.0070329 (PMC3726653; doi:10.1371/journal.pone.0070329)
Supplement: Table S4 — A. Glycosyltransferase genes in the Acinetobacter polysaccharides gene clusters for the 25 PSgc sequence forms. B. Glycosyltransferase genes found in each gene cluster. C. Diversity of shared glycosyltransferase genes. (DOC) [file pone.0070329.s006.doc]

**Table S4A. Glycosyltransferase genes in the *Acinetobacter* polysaccharides gene clusters for the 25 PSgc sequence forms**

| **Gene Name** | **PSgc form** | **GC%** | **Length of AA** | **Pfam^a^** | **CAZy^b^** | **Mechansim^c^** | **Homology group** | **Putative reaction** | **Presence in other PSgc forms** |
| --- | --- | --- | --- | --- | --- | --- | --- | --- | --- |
| *wafA* | PSgc1 | 23.5 | 313 | Glyco_transf_52, PF07922, E=7.7e-44 | 52 | Not known | 17 | N/A | PSgc26 |
| *wafB* | PSgc1 | 30.2 | 358 | Glycos_transf_1, PF00534, E=1.8e-29 | 4 | RET | 11 | N/A | PSgc26 |
| *wafC* | PSgc1 | 32 | 352 | Glycos_transf_1, PF00534, E=6.2e-34 | 4 | RET | 2 | N/A | PSgc26 |
| *wafD* | PSgc2 | 26.9 | 280 | Glycos_transf_2, PF00535, E=1.3e-25 | 2 | INV | 1 | FucNAc(R3Hb)(β1→3)GalNAc | N/A |
| *wafE* | PSgc2 | 28 | 364 | Glycos_transf_1, PF00534, E=1.4e-32 | 4 | RET | 4 | GalNAc(α1→3)GalNAc | N/A |
| *wafF* | PSgc2 | 27.3 | 313 | Glycos_transf_2, PF00535, E=1.1e-38 | 2 | INV | 1 | GalNAc(β1→3)Gal | PSgc11 |
| *wafG* | PSgc2 | 31 | 344 | Glycos_transf_1, PF00534, E=2.5e-39 | 4 | RET | 2 | Gal(α1→6)Gal | PSgc4, PSgc9, PSgc11 |
| *wafH* | PSgc2 | 34.4 | 275 | Glycos_transf_2, PF00535, E=3.9e-22 | 2 | INV | 1 | Gal(β1→3)GalNAc | PSgc3, 4, 9, 11, 12, 18, 22 |
| *wafJ* | PSgc3 | 26.6 | 315 | Glycos_transf_2, PF00535, E=9e-36 | 2 | INV | 1 | N/A | N/A |
| *wafK* | PSgc3 | 27.2 | 363 | Glycos_transf_1, PF00534, E=3.2e-28 | 4 | RET | 2 | N/A | N/A |
| *wafL* | PSgc11 | 26.1 | 315 | Glycos_transf_2, PF00535, E=5e-28 | 2 | INV | 5 | Glc(β1→6)GalNAc | PSgc4 |
| *wafM* | PSgc4 | 25.3 | 327 | Glycos_transf_2, PF00535, E=5.3e-34 | 2 | INV | 5 | N/A | N/A |
| *wafN* | PSgc5 | 28.4 | 392 | Glycos_transf_1, PF00534, E=5.5e-11 | 4 | RET | 18 | FucNAc(α1→4)GalNAcA | N/A |
| *wafO* | PSgc5 | 28.4 | 381 | Glycos_transf_1, PF00534, E=3e-20 | 4 | RET | 19 | GalNAcA(α1→3)FucNAc | N/A |
| *wafP* | PSgc5 | 30.7 | 350 | Glycos_transf_1, PF00534, E=2.1e-13 | 4 | RET | 8 | FucNAc(α1→3)GlcNAc | PSgc24 |
| *wafS* | PSgc6 | 30.7 | 343 | Glycos_transf_1, PF00534, E=1.2e-24 | 4 | RET | 4 | N/A | N/A |
| *wafT* | PSgc6 | 37.8 | 368 | Glycos_transf_1, PF00534, E=7e-31 | 4 | RET | 3 | N/A | PSgc13 |
| *wafV* | PSgc9 | 25.9 | 387 | Glycos_transf_1, PF00534, E=6.9e-11 | 4 | RET | 12 | N/A | N/A |
| *wafW* | PSgc9 | 28.7 | 363 | N/A | N/A | N/A | 13 | N/A | N/A |
| *wafX* | PSgc8 | 26.5 | 302 | Glycos_transf_2, PF00535, E=6.6e-26 | 2 | INV | 10 | N/A | PSgc17 |
| *wafY* | PSgc8 | 33.1 | 329 | Glycos_transf_1, PF00534, E=7.3e-37 | 4 | RET | 2 | N/A | PSgc17 |
| *wafZ* | PSgc8 | 28.1 | 266 | Glycos_transf_2, PF00535, E=3.7e-33 | 2 | INV | 1 | N/A | PSgc17 |
| *wagB* | PSgc8 | 25.8 | 346 | N/A | N/A | N/A | 7 | N/A | PSgc17, PSgc19 |
| *wagC* | PSgc10 | 28.2 | 366 | Glycos_transf_1, PF00534, E=8.7e-09 | 4 | RET | 20 | ManNAc(α1→3)Rha | N/A |
| *wagD* | PSgc10 | 28.8 | 295 | Glycos_transf_2, PF00535, E=9.4e-14 | 2 | INV | 6 | Rha(α1→2)Rha | PSgc19 |
| *wagE* | PSgc10 | 31.3 | 267 | Glycos_transf_2, PF00535, E=9.7e-12 | 2 | INV | 9 | Rha(α1→3)Rha/GlcNAc | PSgc14 |
| *wagJ* | PSgc13 | 29.1 | 351 | Glycos_transf_1, PF00534, E=6.8e-33 | 4 | RET | 4 | N/A | N/A |
| *wagK* | PSgc14 | 31.9 | 287 | Glycos_transf_2, PF00535, E=9.7e-05 | 2 | INV | 15 | N/A | PSgc19 |
| *wagL* | PSgc14 | 30.3 | 361 | Glycos_transf_1, PF00534, E=1.6e-09 | 4 | RET | 14 | N/A | PSgc19 |
| *wagM* | PSgc14 | 30.8 | 299 | Glycos_transf_2, PF00535, E=3.6e-10 | 2 | INV | 6 | N/A | N/A |
| *wagN* | PSgc15 | 28.5 | 297 | Glycos_transf_2, PF00535, E=9.9e-28 | 2 | INV | 1 | N/A | N/A |
| *wagO* | PSgc15 | 33.4 | 367 | Glycos_transf_1, PF00534, E=1.7e-31 | 4 | RET | 4 | N/A | PSgc20 |
| *wagP* | PSgc23 | 31.8 | 393 | Glycos_transf_1, PF00534, E=9.9e-32 | 4 | RET | 3 | Gal(β1→3)GlcNAc | PSgc15, PSgc20, PSgc25 |
| *wagQ* | PSgc23 | 26.1 | 298 | Glycos_transf_2, PF00535, E=1.3e-29 | 2 | INV | 1 | Qui3N(R3Hb)(β1→6)GlcNAc | N/A |
| *wagR* | PSgc23 | 29 | 216 | Gly_transf_sug, PF04488, E=1.1e-13 | 32 | RET | 16 | GlcNAc(α1→4)GalNAc | N/A |
| *wagS* | PSgc23 | 27.4 | 296 | Glycos_transf_2, PF00535, E=2.8e-30 | 2 | INV | 1 | GalNAc(β1→3)Gal | N/A |
| *wagT* | PSgc18 | 25.6 | 333 | N/A | N/A | N/A | 21 | Gal(α1→4)Gal | N/A |
| *wagU* | PSgc18 | 30.1 | 344 | Glycos_transf_1, PF00534, E=2.5e-18 | 4 | RET | 4 | ManNAc(β1→4)Gal | N/A |
| *wagV* | PSgc19 | 29.2 | 300 | N/A | N/A | N/A | 22 | N/A | N/A |
| *wagW* | PSgc19 | 30.8 | 233 | Glycos_transf_2, PF00535, E=5.5e-32 | 2 | INV | 1 | N/A | N/A |
| *wagY* | PSgc20 | 26.8 | 320 | Glycos_transf_2, PF00535, E=2e-31 | 2 | INV | 5 | N/A | N/A |
| *wagZ* | PSgc20 | 28 | 355 | Glycos_transf_1, PF00534, E=6.7e-35 | 4 | RET | 2 | N/A | N/A |
| *wahA* | PSgc21 | 25.4 | 303 | Glycos_transf_2, PF00535, E=7.5e-40 | 2 | INV | 5 | N/A | N/A |
| *wahB* | PSgc21 | 25 | 372 | Glycos_transf_1, PF00534, E=2.2e-31 | 4 | RET | 2 | N/A | N/A |
| *wahC* | PSgc21 | 24.3 | 256 | Glycos_transf_2, PF00535, E=1.3e-09 | 2 | INV | 23 | N/A | N/A |
| *wahD* | PSgc21 | 27.8 | 257 | Glycos_transf_2, PF00535, E=7.4e-43 | 2 | INV | 1 | N/A | N/A |
| *wahG* | PSgc24 | 27.1 | 339 | Glycos_transf_1, PF00534, E=7.1e-11 | 4 | RET | 24 | GlcNAc(α1→3)FucNAc | N/A |
| *wahH* | PSgc25 | 31 | 357 | Glycos_transf_1, PF00534, E=1.7e-40 | 4 | RET | 4 | N/A | N/A |
| *wahI* | PSgc25 | 27.4 | 290 | Glycos_transf_2, PF00535, E=5.4e-28 | 2 | INV | 1 | N/A | N/A |
| *wahJ* | PSgc25 | 24.1 | 318 | Glycos_transf_2, PF00535, E=4.1e-28 | 2 | INV | 5 | N/A | N/A |
| *wahK* | PSgc27 | 27.3 | 361 | Glycos_transf_1, PF00534, E=6.3e-06 | 4 | RET | 25 | N/A | N/A |
| *wahL* | PSgc27 | 33.2 | 367 | Glycos_transf_1, PF00534, E=2.9e-22 | 4 | RET | 3 | N/A | N/A |
| *wahM* | PSgc27 | 34.3 | 413 | Glycos_transf_1, PF00534, E=3.4e-15 | 4 | RET | 8 | N/A | N/A |

a． Pfam family (http://pfam.janelia.org/). “N/A” indicates there isn’t a Pfam family fit the gene.

b． CAZy glycosyltransferase group (http://www.cazy.org/GlycosylTransferases.html). “N/A” indicates there isn’t a group fit the gene.

c． Catalytic Mechanism. “INV”: inversion of the anomeric configuration; “RET”: retention of the anomeric configuration.

**Table S4B. Glycosyltransferase genes found in each gene cluster**

| **PSgc forms** | **Gene Name** | **GC%** | **Length of AA** | **Pfam^a^** | **CAZy^b^** | **Mechansim^c^** | **Homology group** | **Reaction** |
| --- | --- | --- | --- | --- | --- | --- | --- | --- |
| PSgc1 | *wafA* | 23.5 | 313 | Glyco_transf_52, PF07922, E=7.7e-44 | 52 | Not known | 17 | N/A |
|  | *wafB* | 30.2 | 358 | Glycos_transf_1, PF00534, E=1.8e-29 | 4 | RET | 11 | N/A |
|  | *wafC* | 32 | 352 | Glycos_transf_1, PF00534, E=6.2e-34 | 4 | RET | 2 | N/A |
| PSgc2 | *wafD* | 26.9 | 280 | Glycos_transf_2, PF00535, E=1.3e-25 | 2 | INV | 1 | FucNAc(R3Hb)(β1→3)GalNAc |
|  | *wafE* | 28 | 364 | Glycos_transf_1, PF00534, E=1.4e-32 | 4 | RET | 4 | GalNAc(α1→3)GalNAc |
|  | *wafF* | 27.3 | 313 | Glycos_transf_2, PF00535, E=1.1e-38 | 2 | INV | 1 | GalNAc(β1→3)Gal |
|  | *wafG* | 31 | 344 | Glycos_transf_1, PF00534, E=2.5e-39 | 4 | RET | 2 | Gal(α1→6)Gal |
|  | *wafH* | 34.4 | 275 | Glycos_transf_2, PF00535, E=3.9e-22 | 2 | INV | 1 | Gal(β1→3)GalNAc |
| PSgc3 | *wafH* | 33.2 | 276 | Glycos_transf_2, PF00535, E=8.2e-26 | 2 | INV | 1 | N/A |
|  | *wafJ* | 26.6 | 315 | Glycos_transf_2, PF00535, E=9e-36 | 2 | INV | 1 | N/A |
|  | *wafK* | 27.2 | 363 | Glycos_transf_1, PF00534, E=3.2e-28 | 4 | RET | 2 | N/A |
| PSgc4 | *wafG* | 31.8 | 344 | Glycos_transf_1, PF00534, E=1.2e-39 | 4 | RET | 2 | N/A |
|  | *wafH* | 34.2 | 275 | Glycos_transf_2, PF00535, E=9.4e-22 | 2 | INV | 1 | N/A |
|  | *wafL* | 26.4 | 315 | Glycos_transf_2, PF00535, E=4.1e-28 | 2 | INV | 5 | N/A |
|  | *wafM* | 25.3 | 327 | Glycos_transf_2, PF00535, E=5.3e-34 | 2 | INV | 5 | N/A |
| PSgc5 | *wafN* | 28.4 | 392 | Glycos_transf_1, PF00534, E=5.5e-11 | 4 | RET | 18 | FucNAc(α1→4)GalNAcA |
|  | *wafO* | 28.4 | 381 | Glycos_transf_1, PF00534, E=3e-20 | 4 | RET | 19 | GalNAcA(α1→3)LN/AFucNAc |
|  | *wafP* | 30.7 | 350 | Glycos_transf_1, PF00534, E=2.1e-13 | 4 | RET | 8 | FucNAc(α1→3)GlcNAc |
| PSgc6 | *wafS* | 30.7 | 343 | Glycos_transf_1, PF00534, E=1.2e-24 | 4 | RET | 4 | N/A |
|  | *wafT* | 37.8 | 368 | Glycos_transf_1, PF00534, E=7e-31 | 4 | RET | 3 | N/A |
| PSgc8 | *wafX* | 26.5 | 302 | Glycos_transf_2, PF00535, E=6.6e-26 | 2 | INV | 10 | N/A |
|  | *wafY* | 33.1 | 329 | Glycos_transf_1, PF00534, E=7.3e-37 | 4 | RET | 2 | N/A |
|  | *wafZ* | 28.1 | 266 | Glycos_transf_2, PF00535, E=3.7e-33 | 2 | INV | 1 | N/A |
|  | *wagB* | 25.8 | 346 | N/A | N/A | N/A | 7 | N/A |
| PSgc9 | *wafG* | 30.6 | 344 | Glycos_transf_1, PF00534, E=3.2e-38 | 4 | RET | 2 | N/A |
|  | *wafH* | 31 | 275 | Glycos_transf_2, PF00535, E=9.4e-21 | 2 | INV | 1 | N/A |
|  | *wafV* | 25.9 | 387 | Glycos_transf_1, PF00534, E=6.9e-11 | 4 | RET | 12 | N/A |
|  | *wafW* | 28.7 | 363 | N/A | N/A | N/A | 13 | N/A |
| PSgc10 | *wagC* | 28.2 | 366 | Glycos_transf_1, PF00534, E=8.7e-09 | 4 | RET | 20 | ManNAc(α1→3)Rha |
|  | *wagD* | 28.8 | 295 | Glycos_transf_2, PF00535, E=9.4e-14 | 2 | INV | 6 | Rha(α1→2)Rha |
|  | *wagE* | 31.3 | 267 | Glycos_transf_2, PF00535, E=9.7e-12 | 2 | INV | 9 | Rha(α1→3)Rha/GlcNAc |
| PSgc11 | *wafF* | 27.7 | 303 | Glycos_transf_2, PF00535, E=1.1e-36 | 2 | INV | 1 | GalNAc(β1→3)Gal |
|  | *wafG* | 31.7 | 344 | Glycos_transf_1, PF00534, E=1.4e-39 | 4 | RET | 2 | Gal(α1→6)Gal |
|  | *wafH* | 34.5 | 275 | Glycos_transf_2, PF00535, E=1.4e-22 | 2 | INV | 1 | Gal(β1→3)GalNAc |
|  | *wafL* | 26.1 | 315 | Glycos_transf_2, PF00535, E=5e-28 | 2 | INV | 5 | Glc(β1→6)GalNAc |
| PSgc12 | *wafH* | 34.3 | 277 | Glycos_transf_2, PF00535, E=2.3e-23 | 2 | INV | 1 | N/A |
| PSgc13 | *wafT* | 33.4 | 387 | Glycos_transf_1, PF00534, E=2.3e-27 | 4 | RET | 3 | N/A |
|  | *wagJ* | 29.1 | 351 | Glycos_transf_1, PF00534, E=6.8e-33 | 4 | RET | 4 | N/A |
| PSgc14 | *wagE* | 35.5 | 271 | Glycos_transf_2, PF00535, E=3e-14 | 2 | INV | 9 | N/A |
|  | *wagK* | 31.9 | 287 | Glycos_transf_2, PF00535, E=9.7e-05 | 2 | INV | 15 | N/A |
|  | *wagL* | 30.3 | 361 | Glycos_transf_1, PF00534, E=1.6e-09 | 4 | RET | 14 | N/A |
|  | *wagM* | 30.8 | 299 | Glycos_transf_2, PF00535, E=3.6e-10 | 2 | INV | 6 | N/A |
| PSgc15 | *wagN* | 28.5 | 297 | Glycos_transf_2, PF00535, E=9.9e-28 | 2 | INV | 1 | N/A |
|  | *wagO* | 33.4 | 367 | Glycos_transf_1, PF00534, E=1.7e-31 | 4 | RET | 4 | N/A |
|  | *wagP* | 35.6 | 385 | Glycos_transf_1, PF00534, E=2.8e-34 | 4 | RET | 3 | N/A |
| PSgc17 | *wafX* | 24.5 | 300 | Glycos_transf_2, PF00535, E=1e-25 | 2 | INV | 10 | N/A |
|  | *wafY* | 32.6 | 359 | Glycos_transf_1, PF00534, E=9.1e-37 | 4 | RET | 2 | N/A |
|  | *wafZ* | 26.6 | 261 | Glycos_transf_2, PF00535, E=1.8e-35 | 2 | INV | 1 | N/A |
|  | *wagB* | 25.7 | 347 | N/A | N/A | N/A | 7 | N/A |
| PSgc18 | *wafH* | 34.4 | 276 | Glycos_transf_2, PF00535, E=1.4e-22 | 2 | INV | 1 | Gal(β1→3)GalNAc |
|  | *wagT* | 25.6 | 333 | N/A | N/A | N/A | 21 | Gal(α1→4)Gal |
|  | *wagU* | 30.1 | 344 | Glycos_transf_1, PF00534, E=2.5e-18 | 4 | RET | 4 | ManNAc(β1→4)Gal |
| PSgc19 | *wagB* | 27.4 | 343 | N/A | N/A | N/A | 7 | N/A |
|  | *wagD* | 31.5 | 300 | Glycos_transf_2, PF00535, E=4.1e-09 | 2 | INV | 6 | N/A |
|  | *wagK* | 31.8 | 279 | Glycos_transf_2, PF00535, E=9.1e-06 | 2 | INV | 15 | N/A |
|  | *wagL* | 30.2 | 360 | Glycos_transf_1, PF00534, E=5.4e-09 | 4 | RET | 14 | N/A |
|  | *wagV* | 29.2 | 300 | N/A | N/A | N/A | 22 | N/A |
|  | *wagW* | 30.8 | 233 | Glycos_transf_2, PF00535, E=5.5e-32 | 2 | INV | 1 | N/A |
| PSgc20 | *wagO* | 33.6 | 367 | Glycos_transf_1, PF00534, E=9.2e-32 | 4 | RET | 4 | N/A |
|  | *wagP* | 35.2 | 385 | Glycos_transf_1, PF00534, E=2.8e-34 | 4 | RET | 3 | N/A |
|  | *wagY* | 26.8 | 320 | Glycos_transf_2, PF00535, E=2e-31 | 2 | INV | 5 | N/A |
|  | *wagZ* | 28 | 355 | Glycos_transf_1, PF00534, E=6.7e-35 | 4 | RET | 2 | N/A |
| PSgc21 | *wahA* | 25.4 | 303 | Glycos_transf_2, PF00535, E=7.5e-40 | 2 | INV | 5 | N/A |
|  | *wahB* | 25 | 372 | Glycos_transf_1, PF00534, E=2.2e-31 | 4 | RET | 2 | N/A |
|  | *wahC* | 24.3 | 256 | Glycos_transf_2, PF00535, E=1.3e-09 | 2 | INV | 23 | N/A |
|  | *wahD* | 27.8 | 257 | Glycos_transf_2, PF00535, E=7.4e-43 | 2 | INV | 1 | N/A |
| PSgc22 | *wafH* | 34.8 | 276 | Glycos_transf_2, PF00535, E=1.2e-20 | 2 | INV | 1 | N/A |
| PSgc23 | *wagP* | 31.8 | 393 | Glycos_transf_1, PF00534, E=9.9e-32 | 4 | RET | 3 | Gal(β1→3)GlcNAc |
|  | *wagQ* | 26.1 | 298 | Glycos_transf_2, PF00535, E=1.3e-29 | 2 | INV | 1 | Qui3N(R3Hb)(β1→6)GlcNAc |
|  | *wagR* | 29 | 216 | Gly_transf_sug, PF04488, E=1.1e-13 | 32 | RET | 16 | GlcNAc(α1→4)GalNAc |
|  | *wagS* | 27.4 | 296 | Glycos_transf_2, PF00535, E=2.8e-30 | 2 | INV | 1 | GalNAc(β1→3)Gal |
| PSgc24 | *wafP* | 31 | 370 | Glycos_transf_1, PF00534, E=2.4e-13 | 4 | RET | 8 | FucNAc(α1→3)GlcNAc |
|  | *wahG* | 27.1 | 339 | Glycos_transf_1, PF00534, E=7.1e-11 | 4 | RET | 24 | GlcNAc(α1→3)FucNAc |
| PSgc25 | *wagP* | 35.8 | 386 | Glycos_transf_1, PF00534, E=5.6e-35 | 4 | RET | 3 | N/A |
|  | *wahH* | 31 | 357 | Glycos_transf_1, PF00534, E=1.7e-40 | 4 | RET | 4 | N/A |
|  | *wahI* | 27.4 | 290 | Glycos_transf_2, PF00535, E=5.4e-28 | 2 | INV | 1 | N/A |
|  | *wahJ* | 24.1 | 318 | Glycos_transf_2, PF00535, E=4.1e-28 | 2 | INV | 5 | N/A |
| PSgc26 | *wafA* | 22 | 309 | Glyco_transf_52, PF07922, E=1e-40 | 52 | Not known | 17 | N/A |
|  | *wafB* | 30.4 | 358 | Glycos_transf_1, PF00534, E=1.7e-29 | 4 | RET | 11 | N/A |
|  | *wafC* | 31.9 | 352 | Glycos_transf_1, PF00534, E=3.1e-33 | 4 | RET | 2 | N/A |
| PSgc27 | *wahK* | 27.3 | 361 | Glycos_transf_1, PF00534, E=6.3e-06 | 4 | RET | 25 | N/A |
|  | *wahL* | 33.2 | 367 | Glycos_transf_1, PF00534, E=2.9e-22 | 4 | RET | 3 | N/A |
|  | *wahM* | 34.3 | 413 | Glycos_transf_1, PF00534, E=3.4e-15 | 4 | RET | 8 | N/A |

a. Pfam family (http://pfam.janelia.org/). “N/A” indicates there isn’t a Pfam family fit the gene.

b. CAZy glycosyltransferase group (http://www.cazy.org/GlycosylTransferases.html). “N/A” indicates there isn’t a group fit the gene.

c. Catalytic Mechanism. “INV”: inversion of the anomeric configuration; “RET”: retention of the anomeric configuration.

**Table S4C. Diversity of shared glycosyltransferase genes**

| **shared GT** | **minimum identity^a^** | **maximum identity^a^** | **present in PSgc forms** |
| --- | --- | --- | --- |
| *wafH* | 61.82% | 100% | PSgc2, 3, 4, 9, 11, 12, 18, 22 |
| *wafG* | 73.73% | 97.67% | PSgc2, PSgc4, PSgc9, PSgc11 |
| *wagP* | 60.36% | 98.96% | PSgc15, PSgc20, PSgc23, PSgc25 |
| *wagB* | 90.38% | 97.12% | PSgc8, PSgc17, PSgc19 |
| *wafP* | 100% | - | PSgc5, PSgc24 |
| *wafT* | 69.40% | - | PSgc6, PSgc13 |
| *wafA* | 57.54% | - | PSgc1, PSgc26 |
| *wafB* | 96.65% | - | PSgc1, PSgc26 |
| *wafC* | 98.58% | - | PSgc1, PSgc26 |
| *wafL* | 80.39% | - | PSgc4, PSgc11 |
| *wafF* | 97.69% | - | PSgc2, PSgc11 |
| *wagD* | 60.35% | - | PSgc10, PSgc19 |
| *wagE* | 70.45% | - | PSgc10, PSgc14 |
| *wafX* | 80.00% | - | PSgc8, PSgc17 |
| *wafY* | 99.70% | - | PSgc8, PSgc17 |
| *wafZ* | 64.02% | - | PSgc8, PSgc17 |
| *wagK* | 54.09% | - | PSgc14, PSgc19 |
| *wagL* | 85.83% | - | PSgc14, PSgc19 |
| *wagO* | 98.64% | - | PSgc15, PSgc20 |

1. Using Blastp with protein sequences.
