# Supplementary material for: Diversity in the Major Polysaccharide Antigen of Acinetobacter Baumannii Assessed by DNA Sequencing, and Development of a Molecular Serotyping Scheme
Source: PLoS One. 2013 Jul 29;8(7):e70329. doi: 10.1371/journal.pone.0070329 (PMC3726653; doi:10.1371/journal.pone.0070329)
Supplement: Table S5 — wzy genes in the Acinetobacter polysaccharides gene clusters for the 25 PSgc sequence forms. (DOC) [file pone.0070329.s007.doc]

**Table S5. *wzy* genes in the *Acinetobacter* polysaccharides gene clusters for the 25 PSgc sequence forms**

| **PSgc forms** | **GC%** | **Length of AA** | **Homology group** | **Linkage** |
| --- | --- | --- | --- | --- |
| PSgc1 | 21.0 | 353 | 1 | - |
| PSgc2 | 24.2 | 377 | 2 | GalNAc(β1→4) Gal |
| PSgc3 | 27.8 | 408 | 3 | - |
| PSgc4 | 24.4 | 322 | 4 | - |
| PSgc5 | 25.2 | 322 | 5 | GlcNAc(α1→3) GalNAcA |
| PSgc6 | 24.7 | 361 | 6 | - |
| PSgc8 | 25.7 | 344 | 7 | - |
| PSgc9 | 23.2 | 346 | 8 | - |
| PSgc10 | 21.2 | 385 | 9 | GlcNAc(α1→2) LRha |
| PSgc11 | 25.7 | 460 | 10 | GalNAc(α1→4) GalNAc |
| PSgc12 | 25.1 | 326 | 11 | - |
| PSgc13 | 24.8 | 385 | 12 | - |
| PSgc14 | 27.8 | 412 | 13 | - |
| PSgc15 | 26.4 | 455 | 14 | - |
| PSgc17 | 25.4 | 354 | 15 | - |
| PSgc18 | 26.8 | 343 | 16 | GalNAc(β1→3) Gal |
| PSgc19 | 25.9 | 327 | 17 | - |
| PSgc20 | 24.3 | 331 | 18 | - |
| PSgc21 | 23.0 | 365 | 19 | - |
| PSgc22 | 27.1 | 328 | 20 | - |
| PSgc23 | 24.9 | 346 | 21 | GlcNAc(β1→3) GalNAc |
| PSgc24 | 23.8 | 406 | 22 | GlcNAc(α1→4) Leg5Ac7Ac |
| PSgc25 | 25.2 | 355 | 23 | - |
| PSgc26 | 23.5 | 355 | 24 | - |
| PSgc27 | 29.1 | 468 | 25 | - |
