# Supplementary material for: Diversity in the Major Polysaccharide Antigen of Acinetobacter Baumannii Assessed by DNA Sequencing, and Development of a Molecular Serotyping Scheme
Source: PLoS One. 2013 Jul 29;8(7):e70329. doi: 10.1371/journal.pone.0070329 (PMC3726653; doi:10.1371/journal.pone.0070329)
Supplement: Table S6 — Primers used for wzy in Acinetobacter molecular typing. (DOC) [file pone.0070329.s008.doc]

**Table S6. Primers used for *wzy* in *Acinetobacter* molecular typing**

| **PSgc form** | **Target gene** | **Forward/Reverse primers (5'-3')** | **Product size (bp)** |
| --- | --- | --- | --- |
| PSgc1 | *wzy_1* | wl-56554:AGGGCAACCATGCTTTAGAA wl-56555:TTTTTCGACTTTAGCGGATAGA | 191 |
|
| PSgc2 | *wzy_2* | wl-56396:AGAGAATCCCGCGACTAACA wl-56397:TTGTTTGGAATCGGCTTAAA | 195 |
|
| PSgc3 | *wzy_3* | wl-56404:TCAGGGGCGTTTAGTTGGTA wl-56405:ATTACTGTTGTCCGCGAAGC | 186 |
|
| PSgc4 | *wzy_4* | wl-56492:CTGCCACCTGAAAATTCCAT wl-56493:CACAATAGGCTTGGCGATTC | 200 |
|
| PSgc5 | *wzy_5* | wl-56468:CCCACACAAGCCCAAAGTAT wl-56469:GTGAAGGTGCAAAAGGGAAA | 211 |
|
| PSgc6 | *wzy_6* | wl-56420:AAACCCGATACCAGCTTCAA wl-56421:GTCGATCAATCATTGCCACA | 192 |
|
| PSgc8 | *wzy_7* | wl-56436:CCCCGGGAAAATCTGAATAA wl-56437:CAGCCAAGTCAAGCAGAGTG | 200 |
|
| PSgc9 | *wzy_8* | wl-56382:GGCAAGGTTTTGCTATTCCA wl-56383:TGCCACAATAAATCCGAAAA | 236 |
|
|
| PSgc10 | *wzy_9* | wl-56338:TTGCCTTCTGAAACTTTTTGG wl-56339:GCGCCACCATACATTGTTCT | 221 |
|
| PSgc11 | *wzy_10* | wl-56374:GGGTTTGGTTTAGGGTGGTC wl-56375:TGGTGCGAACATAAAAGCAG | 186 |
|
| PSgc12 | *wzy_11* | wl-57215:TCGGGCTCAAAATCTGAAGT wl-57216:AAATTCTTTTTGCTGGAGCATT | 208 |
|
| PSgc13 | *wzy_12* | wl-56460:TGGAATGGAAAAACCAAATGA wl-56461:GATGTTGATATCGGCAGCATT | 190 |
|
| PSgc14 | *wzy_13* | wl-56500:GGGCTTGGTTAAAGGGTGTA wl-56501:GTGCAGTAGTGGCAGCTTCA | 201 |
|
| PSgc15 | *wzy_14* | wl-56366:TGATTTGCACACCAGGAAAA wl-56367:GATTAGTGGGGCACGAGAGA | 170 |
|
|
| PSgc17 | *wzy_15* | wl-56358:AGGAGTCGAACCTGCCTTTT wl-56359:AGCAAGAGCTTGCCTTATCG | 214 |
|
| PSgc18 | *wzy_16* | wl-56444:CAATGTTGAAATACGCCCACT wl-56445:ACTGACGGATCGGATTGGTA | 232 |
|
| PSgc19 | *wzy_17* | wl-56538:TATGGCAATTCGGTATGCAG wl-56539:ATTCACAATGGCTGGAGAGG | 163 |
|
| PSgc20 | *wzy_18* | wl-56476:CAGCTTGCATCTGGGAAACT wl-56477:TGCAAGTGTTTCTGCTCGTT | 176 |
|
| PSgc21 | *wzy_19* | wl-56428:CTGGCGCAATTTTTGATTTT wl-56429:CATAAAATGAGTCAATCCCCAAA | 248 |
|
| PSgc22 | *wzy_20* | wl-56346:GGGTCGAAATCTGAAGTGGA wl-56347:GGTGCACCTGCAAGGAATAA | 191 |
|
| PSgc23 | *wzy_21* | wl-56508:CCCTGAGGGATCAGTTCCTA wl-56509:AAGTTGCTGATAAGAGTGGTGGT | 185 |
|
| PSgc24 | *wzy_22* | wl-56412:ATTCCAAGTGAGAGCCGTGT wl-56413:AACTTAAGCCCACCCCAAAT | 158 |
|
| PSgc25 | *wzy_23* | wl-56546:AATGTTTTCGTCTCCGAAGC wl-56547:AACCGTAAGCTGGTTCCGTA | 185 |
|
| PSgc26 | *wzy_24* | wl-57217:CACGCCGATAAAACGAACTT wl-57218:TTCTACCTTGGTCGATCGATTT | 180 |
|
| PSgc27 | *wzy_25* | wl-56452:GCACCAACACCAGGAGCTAT wl-56453:TTGACCATCATCGTCGAATC | 206 |
|
