# Supplementary material for: Diversity in the Major Polysaccharide Antigen of Acinetobacter Baumannii Assessed by DNA Sequencing, and Development of a Molecular Serotyping Scheme
Source: PLoS One. 2013 Jul 29;8(7):e70329. doi: 10.1371/journal.pone.0070329 (PMC3726653; doi:10.1371/journal.pone.0070329)
Supplement: Table S7 — PSgc allocation of Acinetobacter strains with genome sequences. (DOC) [file pone.0070329.s009.doc]

**Table S7. PSgc allocation of *Acinetobacter* strains with genome sequences**

| **PSgc form** | **Species** | **Strain** | **Sequence type ^a^** | **European clone ^b^** | **Accession No.** |
| --- | --- | --- | --- | --- | --- |
| PSgc1 | baumannii | AB210 | 2 | II | AEOX00000000 |
|  | baumannii | LUH5533 | N/A | N/A | DRS005660 |
|  | baumannii | OIFC035 | N/A | N/A | AMTB00000000 |
|  | baumannii | TCDC_AB0715 | 2 | II | CP002522 |
|  | baumannii | W6976 | 2 | II | AIEG00000000 |
|  | baumannii | W7282 | 2 | II | AIEH00000000 |
| PSgc2 | nosocomialis | LUH3483 | 68 | N/A | DRS005640 |
| PSgc3 | baumannii | LUH5534 | 131 | N/A | DRS005641 |
| PSgc4 | nosocomialis | LUH5536 | 68 | N/A | DRS005652 |
|  | baumannii | Naval_82 | N/A | N/A | AMSW00000000 |
| PSgc5 | baumannii | AB_TG27343 | N/A | N/A | AMIS00000000 |
|  | baumannii | ABNIH2 | 2 | II | AFTA00000000 |
|  | baumannii | D1279779 | N/A | N/A | AERZ00000000 |
|  | baumannii | LUH3484 | 2 | II | DRS005649 |
|  | baumannii | MDR_TJ | 2 | II | CP003500 |
|  | baumannii | OIFC098 | 10 | N/A | AMDF00000000 |
| PSgc6 | baumannii | LUH5535 | N/A | N/A | DRS005643 |
| PSgc8 | baumannii | A118 | N/A | N/A | AEOW00000000 |
|  | baumannii | LUH5538 | 40 | N/A | DRS005645 |
| PSgc9 | baumannii | 3909 | 78 | N/A | AEOZ00000000 |
|  | baumannii | 5711 | 2 | II | AHAJ00000000 |
|  | baumannii | AB_TG19617 | N/A | N/A | AMID00000000 |
|  | baumannii | Ab44444 | 2 | II | AKAT00000000 |
|  | baumannii | ABIsac_ColiR | 2 | II | CAKB00000000 |
|  | baumannii | ABIsac_ColiS | 2 | II | CAKA00000000 |
|  | baumannii | ABNIH1 | N/A | II | AFSZ00000000 |
|  | baumannii | AC12 | 2 | II | ALAM00000000 |
|  | baumannii | AC30 | 2 | II | ALXD00000000 |
|  | baumannii | ATCC_17978 | N/A | N/A | CP000521 |
|  | baumannii | ATCC_19606 | 52 | N/A | ACQB00000000 |
|  | baumannii | IS_143 | N/A | II | AMGE00000000 |
|  | baumannii | LUH5537 | 2 | II | DRS005639 |
|  | baumannii | LUH5539 | 2 | II | DRS005655 |
|  | baumannii | Naval_2 | 2 | II | AMSX00000000 |
|  | baumannii | OIFC032 | 32 | N/A | AFCZ00000000 |
|  | baumannii | OIFC099 | 32 | N/A | AMFT00000000 |
|  | baumannii | WC_348 | N/A | N/A | AMZT00000000 |
| PSgc10 | baumannii | LUH5540 | 40 | N/A | DRS005634 |
| PSgc11 | baumannii | 5256 | 25 | N/A | AHAI00000000 |
|  | baumannii | AB_2008_15_69 | 25 | N/A | AMHN00000000 |
|  | nosocomialis | Ab22222 | 71 | N/A | AKAR00000000 |
|  | nosocomialis | LUH5541 | N/A | N/A | DRS005638 |
|  | baumannii | UMB003 | 25 | N/A | AEPM00000000 |
|  | baumannii | WC_487 | N/A | N/A | AMZR00000000 |
| PSgc12 | baumannii | 3990 | 2 | II | AEOY00000000 |
|  | baumannii | AB_1582_8 | 2 | II | AMHB00000000 |
|  | baumannii | AB_1583_8 | N/A | N/A | AMHC00000000 |
|  | baumannii | AB_1595_8 | 2 | II | AMHE00000000 |
|  | baumannii | AB_1766_8 | 2 | II | AMJO00000000 |
|  | baumannii | AB_2008_23_07_01_7 | 2 | II | AMHR00000000 |
|  | baumannii | AB_2009_04_02_7 | 2 | II | AMHT00000000 |
|  | baumannii | AB_515_8 | 2 | II | AMHU00000000 |
|  | baumannii | AB_908_12 | 2 | II | AMHV00000000 |
|  | baumannii | AB_909_05 | 2 | II | AMIA00000000 |
|  | baumannii | AB_909_14_7 | 2 | II | AMIB00000000 |
|  | baumannii | AB_TG2026 | 2 | II | AMIH00000000 |
|  | baumannii | AB_TG2631 | 2 | II | AMIM00000000 |
|  | baumannii | AB_TG27323 | 2 | II | AMIN00000000 |
|  | baumannii | AB_TG27327 | 2 | II | AMIO00000000 |
|  | baumannii | AB_TG27331 | 2 | II | AMIP00000000 |
|  | baumannii | AB_TG27335 | 2 | II | AMIQ00000000 |
|  | baumannii | Ab11111 | 2 | II | AKAQ00000000 |
|  | baumannii | ACICU | 2 | II | CP000863 |
|  | baumannii | LUH3713 | 216 | N/A | DRS005656 |
|  | baumannii | Naval_113 | 2 | II | AMZU00000000 |
|  | baumannii | OIFC087 | 32 | N/A | AMFS00000000 |
|  | baumannii | OIFC189 | 2 | II | AFDM00000000 |
|  | nosocomialis | RUH2624 | 71 | N/A | ACQF00000000 |
|  | baumannii | WM99c | 2 | II | AERY00000000 |
| PSgc13 | baumannii | AB_TG2031 | N/A | N/A | AMIK00000000 |
|  | baumannii | AB307_0294 | 1 | I | CP001172 |
|  | baumannii | AYE | 1 | I | CU459141 |
|  | baumannii | LUH5542 | 1 | I | DRS005648 |
|  | baumannii | Naval_83 | 20 | I | AMFK00000000 |
|  | baumannii | OIFC074 | 19 | I | AMDE00000000 |
|  | baumannii | OIFC109 | 3 | III | ALAL00000000 |
|  | baumannii | OIFC137 | 3 | III | AFDK00000000 |
|  | baumannii | TG19582 | 1 | I | AMIV00000000 |
| PSgc14 | baumannii | LUH5544 | N/A | N/A | DRS005653 |
| PSgc15 | baumannii | LUH5544 | 138 | N/A | DRS005637 |
| PSgc17 | baumannii | AB_2007_16_25_01_7 | 241 | N/A | AMHI00000000 |
|  | baumannii | AB_2007_16_27_01 | 241 | N/A | AMHJ00000000 |
|  | baumannii | AB_TG27339 | 241 | N/A | AMIR00000000 |
|  | baumannii | AB900 | 49 | N/A | ABXK00000000 |
|  | baumannii | LUH5545 | N/A | N/A | DRS005636 |
|  | baumannii | OIFC111 | 49 | N/A | AMFY00000000 |
| PSgc18 | baumannii | LUH5546 | N/A | N/A | DRS005646 |
| PSgc19 | baumannii | LUH5547 | N/A | N/A | DRS005658 |
| PSgc20 | baumannii | LUH5548 | N/A | N/A | DRS005650 |
| PSgc21 | baumannii | Ab33333 | N/A | N/A | AKAS00000000 |
|  | baumannii | LUH5549 | N/A | N/A | DRS005644 |
| PSgc22 | baumannii | LUH5550 | 238 | N/A | DRS005635 |
| PSgc23 | baumannii | LUH3712 | N/A | N/A | DRS005654 |
|  | baumannii | LUH3714 | N/A | N/A | DRS005651 |
| PSgc24 | baumannii | LUH5551 | 109 | N/A | DRS005642 |
| PSgc25 | baumannii | LUH5552 | N/A | N/A | DRS005659 |
| PSgc26 | baumannii | LUH5553 | N/A | N/A | DRS005657 |
| PSgc27 | baumannii | Canada_BC_5 | 1 | I | AFDN00000000 |
|  | baumannii | Canada_BC1 | 1 | I | AMSZ00000000 |
|  | baumannii | LUH5554 | N/A | N/A | DRS005647 |
|  | baumannii | Naval_21 | 19 | I | AMSY00000000 |
|  | baumannii | OIFC110 | N/A | N/A | AMFL00000000 |
|  | baumannii | WC_692 | N/A | N/A | AMGG00000000 |
| PSgc39 | baumannii | AB_908_13 | 1 | I | AMHW00000000 |
|  | baumannii | AB_909_02_7 | 1 | I | AMHZ00000000 |
|  | baumannii | AB0057 | 1 | I | CP001182 |
| PSgc40 | baumannii | 1656_2 | 2 | II | CP001921 |
|  | baumannii | AB_2008_15_45 | N/A | II | AMHL00000000 |
|  | baumannii | AB_2008_15_70 | N/A | II | AMHO00000000 |
|  | baumannii | AB_908_14_7 | 2 | II | AMHX00000000 |
|  | baumannii | ABNIH3 | N/A | II | AFTB00000000 |
| PSgc41 | baumannii | AB_1536_8 | N/A | N/A | AMHA00000000 |
|  | baumannii | AB_1594_8 | N/A | N/A | AMHD00000000 |
|  | baumannii | AB_2008_15_52 | N/A | N/A | AMHM00000000 |
|  | baumannii | AB_2008_15_71 | N/A | N/A | AMHP00000000 |
|  | baumannii | AB_2008_23_01_01_7 | N/A | N/A | AMHQ00000000 |
|  | baumannii | AB_2009_04_01_7 | N/A | N/A | AMHS00000000 |
|  | baumannii | AB_909_01_7 | N/A | N/A | AMHY00000000 |
|  | baumannii | AB_TG2018 | N/A | N/A | AMIE00000000 |
|  | baumannii | AB058 | 20 | I | ADHA00000000 |
| PSgc42 | baumannii | AB_1649_8 | 113 | N/A | AMHF00000000 |
|  | baumannii | AB_1650_8 | 113 | N/A | AMHG00000000 |
| PSgc43 | baumannii | 5075 | 1 | I | AHAH00000000 |
|  | baumannii | IS_235 | 1 | I | AMEI00000000 |
|  | baumannii | IS_251 | 1 | I | AMEJ00000000 |
|  | baumannii | IS_58 | 1 | I | AMGH00000000 |
| PSgc44 | baumannii | AB_2008_15_34_7 | 2 | II | AMHK00000000 |
|  | baumannii | Naval_17 | 2 | II | AFDO00000000 |
|  | baumannii | Naval_18 | 25 | N/A | AFDA00000000 |
|  | baumannii | OIFC180 | 2 | II | AMDQ00000000 |
|  | baumannii | UMB001 | 2 | II | AEPK00000000 |
| PSgc45 | baumannii | IS_116 | 136 | N/A | AMGF00000000 |
|  | baumannii | OIFC065 | 136 | N/A | AMFV00000000 |
| PSgc46 | baumannii | SDF | 17 | N/A | CU468230 |
| PSgc47 | baumannii | ZWS1122 | 2 | II | AMGR00000000 |
|  | baumannii | ZWS1219 | 2 | II | AMGS00000000 |
| PSgc48 | pittii | TG6411 | N/A | N/A | AMJI00000000 |
|  | baumannii | WC_136 | 64 | N/A | AMST00000000 |
| PSgc49 | nosocomialis | NCTC_8102 | 74 | N/A | AIEJ00000000 |
|  | nosocomialis | TG21145 | 74 | N/A | AMJH00000000 |
|  | baumannii | UMB002 | 16 | N/A | AEPL00000000 |
| PSgc50 | baumannii | AB_2007_09_110_01_7 | N/A | N/A | AMHH00000000 |
|  | nosocomialis | TG19596 | N/A | N/A | AMIZ00000000 |
| PSgc51 | baumannii | BZICU_2 | 218 | N/A | ALOH00000000 |
| PSgc52 | baumannii | MDR_ZJ06 | 2 | II | CP001937 |
| PSgc53 | baumannii | Naval_72 | N/A | N/A | AMFI00000000 |
| PSgc54 | baumannii | OIFC143 | 25 | N/A | AFDL00000000 |
| PSgc55 | baumannii | OIFC0162 | N/A | N/A | AMFH00000000 |
| PSgc56 | baumannii | TYTH_1 | 2 | II | CP003856 |
| PSgc57 | baumannii | WC_141 | N/A | N/A | AMSS00000000 |
| PSgc58 | N/A | 528 | 119 | N/A | AMJL00000000 |
|  | N/A | TG2027 | 119 | N/A | AMJN00000000 |
|  | N/A | TG27347 | 119 | N/A | AMIT00000000 |
| PSgc59 | haemolyticus | ATCC_19194 | N/A | N/A | ADMT00000000 |
|  | baumannii | IS_123 | 3 | III | ALII00000000 |
|  | haemolyticus | TG19599 | N/A | N/A | AMJA00000000 |
| PSgc60 | N/A | ATCC_27244 | N/A | N/A | ABYN00000000 |
|  | haemolyticus | TG21157 | N/A | N/A | AMJC00000000 |
| PSgc61 | johnsonii | TG19605 | N/A | N/A | AMJD00000000 |
|  | johnsonii | TG19625 | N/A | N/A | AMJE00000000 |
| PSgc62 | N/A | ADP1 | N/A | N/A | CR543861 |
|  | ursingii | DSM_16037 | N/A | N/A | AIEA00000000 |
|  | baylyi | TG19579 | N/A | N/A | AMIC00000000 |
| PSgc63 | N/A | DR1 | N/A | N/A | CP002080 |
|  | calcoaceticus | PHEA_2 | N/A | N/A | CP002177 |
| PSgc64 | bereziniae | LMG_1003 | N/A | N/A | AIEI00000000 |
| PSgc65 | calcoaceticus | RUH2202 | 92 | N/A | ACPK00000000 |
| PSgc66 | calcoaceticus | TG19585 | 60 | N/A | AMIW00000000 |
| PSgc67 | calcoaceticus | TG19588 | 62 | N/A | AMIX00000000 |
| PSgc68 | calcoaceticus | TG19593 | N/A | N/A | AMIY00000000 |
| PSgc69 | N/A | GG2 | N/A | N/A | ALOW00000000 |
| PSgc70 | haemolyticus | TG19602 | N/A | N/A | AMJB00000000 |
| PSgc71 | johnsonii | SH046 | N/A | N/A | ACPL00000000 |
| PSgc72 | junii | SH205 | N/A | N/A | ACPM00000000 |
| PSgc73 | lwoffii | NCTC_5866 | N/A | N/A | AIEL00000000 |
| PSgc74 | lwoffii | SH145 | N/A | N/A | ACPN00000000 |
| PSgc75 | lwoffii | WJ10621 | N/A | N/A | AFQY00000000 |
| PSgc76 | N/A | NBRC_100985 | N/A | N/A | BAEB00000000 |
| PSgc77 | N/A | NCTC_7422 | 69 | N/A | AIED00000000 |
| PSgc78 | N/A | P8_3_8 | N/A | N/A | AFIE00000000 |
| PSgc79 | pittii | D499 | 63 | N/A | AGFH00000000 |
| PSgc80 | radioresistens | DSM_6976___NBRC_102413 | N/A | N/A | AIDZ00000000 |
| PSgc81 | radioresistens | SH164 | N/A | N/A | ACPO00000000 |
| PSgc82 | radioresistens | SK82 | N/A | N/A | ACVR00000000 |
| PSgc83 | radioresistens | TG02010 | N/A | N/A | AMJJ00000000 |
| PSgc84 | radioresistens | WC_A_157 | N/A | N/A | ALIR00000000 |
| PSgc85 | schindleri | TG19614 | N/A | N/A | AMJK00000000 |
| PSgc86 | N/A | SH024 | 93 | N/A | ADCH00000000 |
| PSgc87 | N/A | TG19627 | N/A | N/A | AMJM00000000 |
| PSgc88 | venetianus | VE_C3 | N/A | N/A | ALIG00000000 |
| PSgc89 | lwoffii | TG19636 | N/A | N/A | AMJG00000000 |
| PSgc90 | venetianus | RAG_1___CIP_110063 | N/A | N/A | AKIQ00000000 |
| N/A**^c^** | baumannii | 4190 | 25 | N/A | AEPA00000000 |
|  | baumannii | 4857 | 3 | III | AHAG00000000 |
|  | N/A | 6013113 | 81 | I | ACYR00000000 |
|  | N/A | 6013150 | 81 | I | ACYQ00000000 |
|  | N/A | 6014059 | 2 | II | ACYS00000000 |
|  | baumannii | AB_TG2022 | 2 | II | AMIF00000000 |
|  | baumannii | AB_TG2023 | 2 | II | AMIG00000000 |
|  | baumannii | AB_TG2028 | N/A | N/A | AMII00000000 |
|  | baumannii | AB_TG2030 | N/A | N/A | AMIJ00000000 |
|  | baumannii | AB_TG2032 | N/A | N/A | AMIL00000000 |
|  | baumannii | AB_TG5064 | 2 | II | AMIU00000000 |
|  | baumannii | AB056 | 1 | I | ADGZ00000000 |
|  | baumannii | AB059 | 1 | I | ADHB00000000 |
|  | baumannii | ABNIH4 | 2 | II | AFTC00000000 |
|  | parvus | DSM_16617 | N/A | N/A | AIEB00000000 |
|  | pittii | DSM_21653 | 63 | N/A | AIEK00000000 |
|  | calcoaceticus | DSM_30006 | 62 | N/A | AIEC00000000 |
|  | pittii | DSM_9306 | 64 | N/A | AIEF00000000 |
|  | N/A | HA | N/A | N/A | AJXD00000000 |
|  | baumannii | Naval_13 | 3 | III | AMDR00000000 |
|  | baumannii | Naval_81 | 3 | III | AFDB00000000 |
|  | N/A | NCTC_10304 | 54 | N/A | AIEE00000000 |
|  | junii | TG19608 | N/A | N/A | AMJF00000000 |
|  | baumannii | WC_323 | N/A | N/A | AMZS00000000 |
|  | baumannii | WC_A_694 | 3 | III | AMTA00000000 |

**a. "N/A" indicates the Sequence type of the strain isn’t found in the MLST database.**

**b. "N/A" indicates the strain is not one of EC I, II or III.**

**c. Not applicable as gene cluster absent or fragmented.**
